# Targeting long-chain acylcarnitine accumulation to protect cardiac mitochondrial homeostasis after complete revascularization

**DOI:** 10.1016/j.xcrm.2025.102507

**Published:** 2025-12-16

**Authors:** Rui Lin, Yuyu Li, Shiwei Yang, Hai Gao, Fengjuan Li, Xue Wang, Xin Tan, Zhengkai Wang, Weiyao Chen, Lu Ren, Xiujie Wang, Li Wang, Jun Qin, Wenjie Yin, Jie Du, Yuan Wang

**Affiliations:** 1 Beijing Anzhen Hospital, Capital Medical University, Beijing Institute of Heart Lung and Blood Vessel Diseases, The Key Laboratory of Remodeling-Related Cardiovascular Diseases, Ministry of Education, Beijing 100029, China; 2 Key Laboratory of Genetic Networks, Institute of Genetics and Developmental Biology, Chinese Academy of Sciences, Beijing 100101, China; 3 State Key Laboratory of Cardiovascular Disease, Fuwai Hospital, National Center for Cardiovascular Diseases, Beijing 100037, China; 4 State Key Laboratory of Proteomics, Beijing Proteome Research Center, National Center for Protein Sciences (The PHOENIX Center, Beijing), Beijing 102206, China; 5 Department of Hypertension, The First Hospital of Shanxi Medical University, Shanxi 030000, China

**Keywords:** non-culprit lesion, complete revascularization, reperfusion injury, CPT1A, long-chain acylcarnitine metabolism, hemodynamic shear stress

## Abstract

Approximately 20% of acute myocardial infarction (AMI) patients with multivessel disease experience adverse outcomes after complete revascularization. We aim to investigate the underlying metabolic mechanism of ischemia-reperfusion injury responsible for abnormal hemodynamic stresses in high-risk patients undergoing complete revascularization. Elevated preoperative serum levels of long-chain acylcarnitine (LCAC) 16:1 are associated with an increased risk of poor prognosis following complete revascularization. Multi-omics analyses reveal that reperfusion injury activates fatty acid degradation, and carnitine palmitoyltransferase 1A (CPT1A) is identified as a key regulator of LCACs in the interaction network in porcine models. In the early stages of reperfusion injury in non-culprit lesions, the release and prolonged elevation of circulating LCACs primarily depend on the activation of endothelial CPT1A through hemodynamic injury, which can be reduced using an inhibitor (etomoxir). Excess LCACs enter cardiomyocytes via the organic cation transporter 2, leading to imbalanced mitochondrial quality control and causing cardiomyocyte death.

## Highlights

• LCAC C16:1 as a prognostic biomarker after complete revascularization
• Hemodynamic injury activates endothelial CPT1A-LCAC metabolic pathway
• Excess LCACs uptake through OCTN2 disrupts cardiac mitochondrial quality control
• Etomoxir reduces LCAC accumulation to ameliorate post-reperfusion cardiac remodeling

## Graphical abstract

**Figure:**
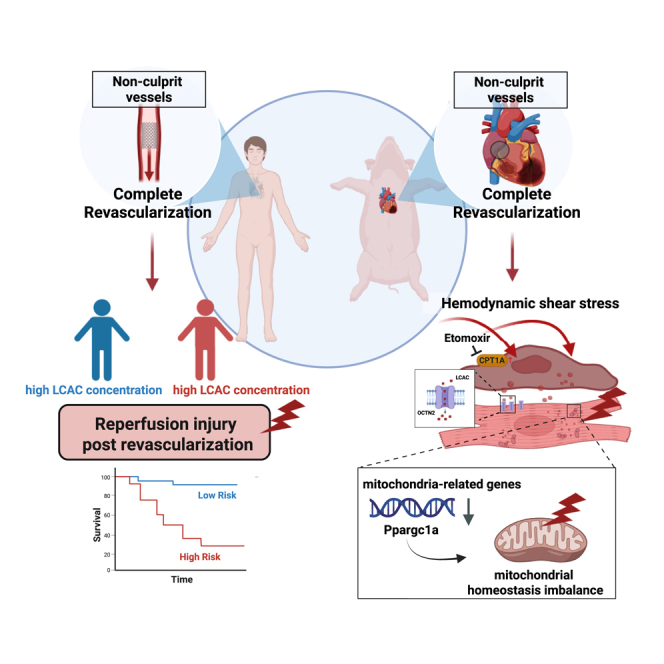

## Teaser

Lin et al. reveal that metabolic dysregulation of long-chain acylcarnitines (LCACs) is a key factor underlying adverse outcomes after complete revascularization in acute myocardial infarction (AMI) patients with multivessel disease. Targeting LCAC accumulation may help prevent reperfusion-induced injury in high-risk AMI patients.

## Introduction

More than 50% of patients with acute myocardial infarction (AMI) have multivessel disease.^1^ In addition to treating culprit lesions, current treatments involve timely opening of non-culprit lesions to reduce adverse cardiovascular events and the risk of reinfarction after recanalization.^1^^,^^2^ The latest research from MULTISTARS-AMI showed that immediate multivessel percutaneous coronary intervention (PCI) was not inferior to staged multivessel PCI with respect to the risk of major adverse cardiovascular events (MACEs).^3^ However, 18% of patients with multivessel disease experienced a serious adverse event after complete revascularization, including heart failure or cardiac death.^4^ Myocardial ischemia-reperfusion (MI/R) injury is a major cause of adverse outcomes after revascularization for myocardial infarction, and evidence suggests that reperfusion through revascularization can trigger a series of events that accelerate reperfusion injury.^5^ Various comorbidities have been implicated in the variable responses of individuals to MI/R injury, ultimately leading to differing degrees of cardiac damage.^6^ However, the clinical background of multivessel lesions is complex. Moreover, there is currently a lack of effective approaches to identify high-risk patients and possible targets for reperfusion injury in non-culprit lesions, which remain a major unmet clinical need.^7^^,^^8^

As with all clinical challenges, advances in basic research using animal models lay the foundation for novel therapeutic approaches that ultimately drive clinical trials.^9^ Patients with multivessel disease are a heterogeneous population, and animal models help to mitigate the influence of confounding clinical factors, enabling the identification of key molecular drivers of injury. To date, research on the mechanism of MI/R injury has mainly been based on small animal experiments, which cannot simulate the complex situation of multiple vessel injuries.^10^ The cardiac size, coronary artery structure, and hemodynamics of miniature pigs closely resemble those of humans, making them a suitable model for accurately studying the characteristics of non-culprit vessel reperfusion injury.^11^^,^^12^

MI/R injury is a complex process that involves multiple metabolic pathways and pathological processes that can lead to cardiac restructuring and dysfunction. Previous studies have been focused on the mechanisms responsible for reperfusion of single culprit vessel lesion.^13^ However, whether there are unique mechanisms responsible for reperfusion injury of non-culprit lesion reopening in multivessel patients remains completely unknown. This damage involves various pathophysiological mechanisms, including mitochondrial energy metabolism, hemodynamic damage, and endothelial dysfunction. Abnormal glucose and lipid metabolism, along with dysregulation of energy supply, are key metabolic pathways contributing to cardiac injury following MI/R.^14^^,^^15^^,^^16^ Mitochondrial energy metabolism and function regulates mitochondrial quality control, which is crucial for maintaining mitochondrial homeostasis. Mitochondrial quality control is primarily mediated by mitochondrial turnover and repair through mitochondrial fission/fusion and mitophagy.^17^ The pathological changes caused by MI/R injury after simultaneous revascularization are characterized by hemodynamic damage, particularly shear stress, which further exacerbates myocardial damage.^18^ Vascular endothelial cells detect and respond to hemodynamic shear stress, and their interactions with cardiomyocytes have been shown to exert effect on MI/R injury.^19^

We performed metabolomics profiling in plasma of individuals to identify LCACs associated with poor outcome after complete revascularization in multicenter cohort study. The level of LCAC C16:1 prior to revascularization was an independent risk factor for adverse cardiovascular events following complete revascularization. Porcine models of AMI with multivessel disease were also developed to simulate reperfusion injury in non-culprit lesions. In a large animal model, LCACs were the most significantly altered fatty acid metabolites in the infarcted myocardium and coronary veins following revascularization of non-culprit lesions. Through a protein–metabolite interaction network, carnitine palmitoyltransferase 1A (CPT1A) was identified as a key regulatory target in LCACs metabolism. Etomoxir (ETO) is a well-characterized, potent, and irreversible inhibitor of CPT1. By inhibiting CPT1, ETO effectively blocks the primary pathway for long-chain fatty acid uptake into the mitochondria, thereby severely impairing mitochondrial fatty acid oxidation.^20^ The application of ETO or LCAC supplementation before reperfusion either improved or worsened acute myocardial mitochondrial dysfunction and long-term myocardial remodeling. The early release of LCACs during non-culprit vessel reperfusion was primarily dependent on the level of CPT1A in endothelial cells, a process that was activated by shear stress or inhibited by ETO. Excess LCACs entered myocardial cells via organic cation transporter 2 (OCTN2), leading to mitochondrial dysfunction and worsening myocardial injury by disrupting mitochondrial quality control pathways.

## Results

### Excessive LCAC elevation was associated with a poor prognosis in patients with AMI with multivessel disease

**Figure 1:**
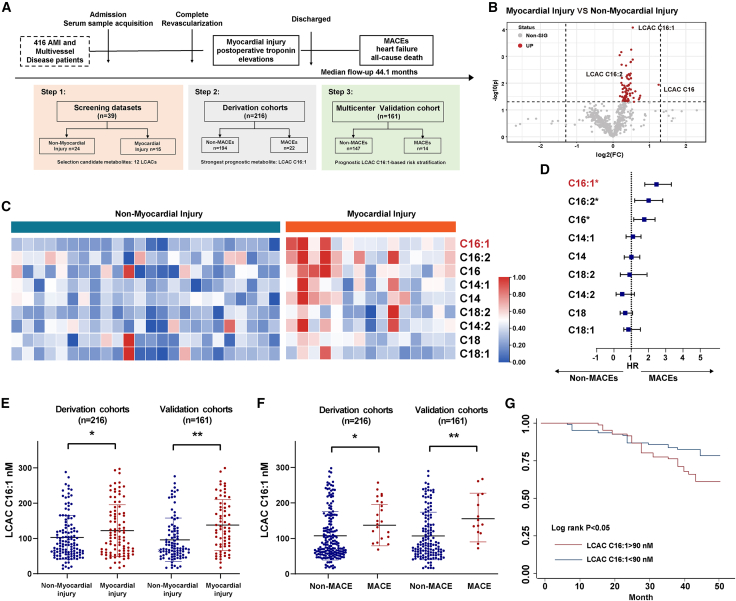
Elevated LCAC concentrations were associated with MACEs in patients with acute myocardial infarction and multivessel disease (A) Enrollment and analysis process of patients with myocardial infarction and multivessel disease. (B) Untargeted metabolomic profiling of plasma from patients in the screening datasets (*n* = 39). Volcano plots highlighted the serum metabolites that increased (red) in myocardial injury, as compared to non-myocardial injury group. (C) Heatmap of LCACs in myocardial injury and non-myocardial injury groups in the screening datasets (*n* = 39); metabolites were rank ordered by fold change. (D) Forest plot of multivariable Cox proportional hazard model for LCACs in derivation cohorts (*n* = 216).Hazard ratio (HR) and *p* value calculated by a Cox proportional hazards model. ∗*p* < 0.05. (E and F) Concentrations of LCAC C16:1 in myocardial injury and non-myocardial injury/MACE and non-MACE groups in derivation cohorts (*n* = 216) or validation cohorts (*n* = 161), respectively. Data were presented as means ± standard deviations as indicated. Data were compared by unpaired Student’s *t* test. ∗*p* < 0.05; ∗∗*p* < 0.01. (G) Kaplan-Meier curves of LCAC C16:1 in derivation cohorts and validation cohorts (*n* = 377). MACEs, adverse cardiovascular events; LCAC, long-chain acylcarnitine.

Metabolomics assays were used to identify metabolites associated with poor outcomes in a multicenter cohort study (BIOMS-IHD). The screening dataset comprised 39 patients, the derivation cohort consisted of 216 patients, and the validation cohort consisted of 161 patients. In the derivation cohort, 36 patients (16.67%) exhibited a poor prognosis, while in the validation cohort, 23 patients (14.28%) experienced similar events (Figure 1A; Tables S1 and S2). In the two cohorts, patients in the group with MACEs were significantly more likely to have diabetes mellitus, hypertension, and higher Global Registry of Acute Coronary Events (GRACE) scores or Synergy between Percutaneous Coronary Intervention with Taxus and Cardiac Surgery (SYNTAX) scores (all *p* < 0.05). The volcano plot shows that the concentrations of multiple LCACs increased through untargeted metabolomics analysis in the screening datasets (Figure 1B). LCAC 16:1 exhibited the most significant upregulation in the myocardial injury group in the screening dataset (Figure 1C). A correlation analysis showed that LCAC C16:1 concentrations were strongly correlated with clinical parameters including glycated hemoglobin, fasting blood sugar concentrations, and diastolic blood pressure in all cohorts (correlation coefficient: r > 0.5, *p* < 0.05) (Figure S1). Thus, the multivariable Cox proportional hazards model was used to assess the association of LCACs with MACEs after adjusting for potential confounders, including diabetes, hyperlipidemia, fasting blood glucose, triglyceride levels, and total cholesterol levels, in the derivation cohort (hazard ratio 2.32) (Figure 1D). LCAC C16:1 was upregulated in the groups with myocardial injury and MACEs in the derivation and validation cohorts (Figures 1E and 1F). The median LCAC 16:1 concentration was used as the cutoff to delineate high (>90 nM) and low (<90 nM) concentrations. The Kaplan-Meier analysis showed that a high LCAC C16:1 concentration was associated with a poor patient prognosis (log rank *p* < 0.05) (Figure 1G). Area under the curve statistics indicated that the combination of LCAC C16:1 with the SYNTAX or GRACE score demonstrates better predictive performance than SYNTAX (area under the receiver operating characteristic curve [AUC] increases 0.10, *p* < 0.05) or GRACE (AUC increases 0.09, *p* < 0.05) (Table S3).

### The sustained increase in LCAC depended on endothelial CPT1A stimulation by shear stress in the large animal model

**Figure 2:**
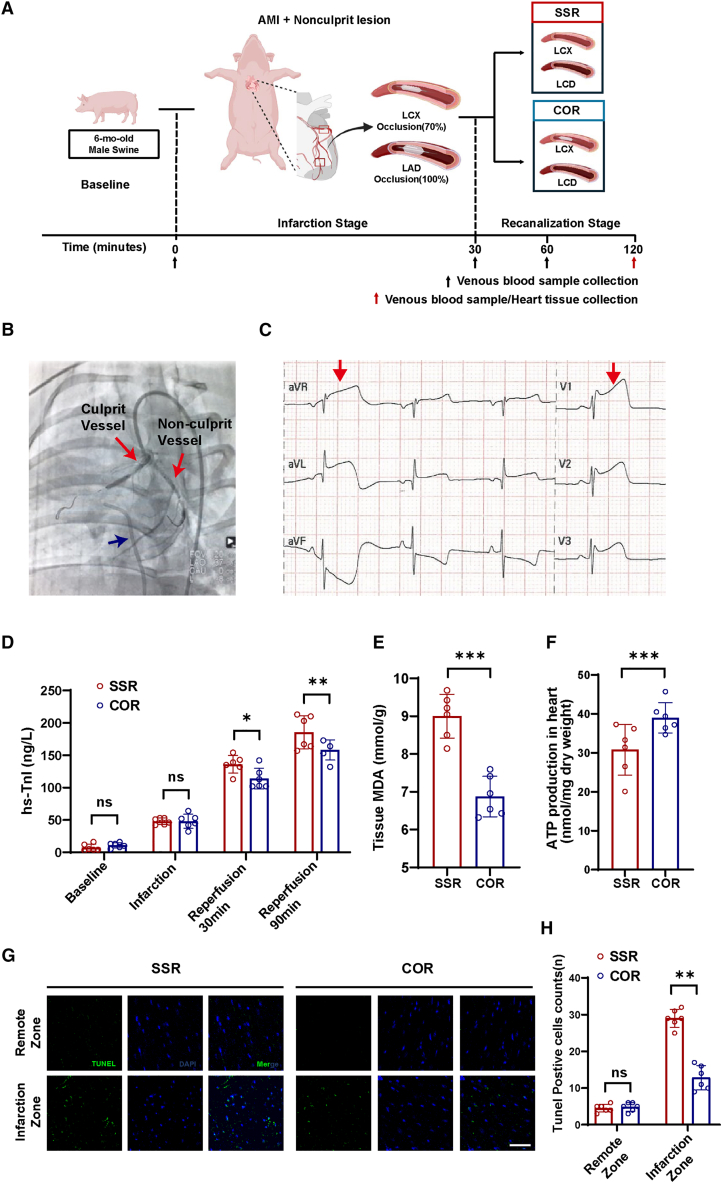
Construction of a swine model of acute myocardial infarction with multivessel coronary disease to simulate reperfusion injury in non-culprit lesions (A) Flowchart of multivessel disease swine model to mimic reperfusion injury in non-culprit lesion. (B) Angiography shows that the model was successfully constructed. The red arrow on the left shows the culprit vessel, while the arrow on the right shows the non-culprit artery. The blue arrow on the left shows collected coronary vein blood. (C) Electrocardiogram at 30 min of infarction. (D) Serum high-sensitivity troponin concentrations were measured at baseline, 30 min after infarction, and at 30 and 90 min after reperfusion in both the SSR and COR groups (*n* = 6/group). Data were presented as means ± standard deviations as indicated. ∗*p* < 0.05 and ∗∗*p* < 0.01 in a Student’s unpaired *t* test. (E) Malondialdehyde levels after recanalization in the SSR and COR groups (*n* = 6/group). Data were presented as means ± standard deviations as indicated. ∗∗∗*p* < 0.001 in a Student’s unpaired *t* test. (F) ATP production in heart tissue after recanalization in the SSR and COR groups (*n* = 6/group). Data were presented as means ± standard deviations as indicated. ∗∗∗*p* < 0.001 in a Student’s unpaired *t* test. (G) Representative images of TUNEL staining of heart tissue after recanalization in the SSR and COR groups. Scale bar: 50 μm. (H) Ratio of TUNEL-positive cells (%) in the SSR and COR groups after recanalization. Data were presented as means ± SD as indicated. Data were compared by unpaired Student’s *t* test (*n* = 6/group); ∗∗*p* < 0.01 in a Student’s unpaired *t* test. LAD, left anterior descending artery; LCX, left circumflex artery; SSR, single-stage revascularization group; COR, culprit-only revascularization group; hs-TnI, high-sensitivity troponin I; MDA, malondialdehyde; ATP, adenosine triphosphate.

To determine the causative role of LCAC in poor prognosis of non-culprit lesion opening and mechanisms involved, a large animal model with multivessel disease was developed to simulate reperfusion injury in non-culprit lesions. Angiography, electrocardiography, and serum high-sensitivity troponin concentrations confirmed the successful establishment of AMI. Coronary angiography revealed complete occlusion of the left anterior descending branch (culprit vessel) and approximately 70% occlusion of the left circumflex branch (non-culprit artery) (Figure 2B). AMI was confirmed by ST-segment elevation on electrocardiogram (Figure 2C). The concentration of serum high-sensitivity troponin from the coronary venous circulation was upregulated during ischemia and reperfusion (*p* < 0.05). Furthermore, the concentration of high-sensitivity troponin in the single-stage revascularization (SSR) group was significantly higher than in the control group (*p* < 0.05) (Figure 2D). Malondialdehyde and adenosine triphosphate (ATP) levels in the infarcted heart were significantly higher in the control group than in the SSR group (*p* < 0.05) (Figures 2E and 2F). The level of apoptosis in the infarcted area was significantly higher in the SSR group than in the control group (*p* < 0.05) (Figures 2G and 2H). Hematoxylin and eosin staining showed that the degree of the inflammatory response was not significantly different between the SSR and control groups (Figure S3A). The data indicate significant mitochondrial dysfunction in the myocardium and cardiomyocyte apoptosis resulting from reperfusion in non-culprit lesions.

**Figure 3:**
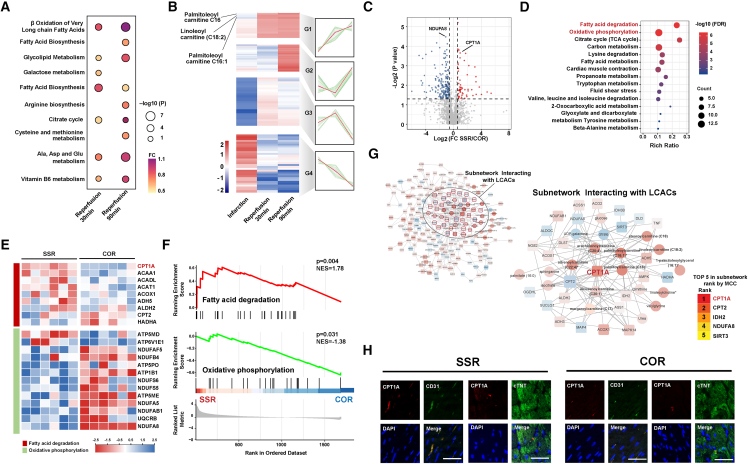
Multi-omics data to identify regulator of the LCAC response to reperfusion injury (A) KEGG metabolic pathway enrichment of differential metabolites (SSR vs. COR) of coronary veins in after 30 and 90 min of reperfusion (*n* = 6/group). (B) Trend expression of differential metabolites of coronary veins at baseline, 30 min after infarction, and at 30 and 90 min after reperfusion in SSR group (*n* = 6/group). (C) Volcano plot of differential proteins (SSR vs. COR) in infarcted heart tissue (*n* = 6/group). (D) KEGG pathway enrichment analysis of differential proteins (SSR vs. COR). (E) Heatmap of differential proteins of fatty acid degradation and the oxidative phosphorylation pathways in the SSR and COR groups (*n* = 6/group). (F) GSEA of fatty acid degradation and the oxidative phosphorylation (SSR vs. COR). (G) Protein-metabolism interaction network and subnetwork interacting with LCACs. (H) Immunostaining for CD31/cTNT (green) and CPT1A (red) on infarcted cardiac tissue sections from the SSR and COR groups. Scale bar, 50 μm. KEGG, Kyoto Encyclopedia of Genes and Genomes; SSR, single-stage revascularization group; COR, culprit-only revascularization group; FC, fold change; GSEA, gene set enrichment analysis; NES, normalized enrichment score; LCAC, long-chain acylcarnitine; MCC, maximal clique centrality.

Proteomics and metabolomics analyses of the plasma samples and cardiac tissues from the swine model of AMI with acute reperfusion injury in non-culprit lesions are shown in Figure S2A. Kyoto Encyclopedia of Genes and Genomes (KEGG) functional enrichment analysis was conducted to detect differential circulating metabolites between the SSR and control groups after 30 and 90 min of reperfusion. β-Lipid oxidation gradually increased after 90 min of reperfusion, suggesting that lipid degradation progressively increased following reperfusion (Figure 3A). When the differential metabolites were grouped according to the trend in their metabolic changes using series test of cluster in the SSR group, the metabolites could be divided into four groups: G1, G2, G3, and G4 (Figure 3B). Among them, the metabolites in group G1 continued to increase after 30 and 90 min of recanalization, with the most significant changes observed for LCACs C16 and C18:2. After 30 and 90 min of reperfusion, two LCACs (LCAC 16 and LCAC 18:2) were upregulated in the SSR group compared with the control group (*p* < 0.05) (Figure S2B). As shown in the volcano plot, seven types of LCACs were upregulated in the SSR group compared with the control group (fold change > 1.2) (Figure S2C). Among the top 15 metabolites ranked by their variable importance in projection scores, 10 were included as LCACs in the orthogonal partial least squares discriminant analysis (Figure S2D). This discovery suggested that recanalization of non-culprit lesions can lead to sustained LCACs elevation.

Differential expression analysis of the proteome showed that after reopening non-culprit lesions, a series of protein changes occurred in the infarcted myocardium, with 419 differentially expressed proteins (Figure 3C). The KEGG enrichment analysis showed that multiple metabolic pathways were involved after recanalization of non-culprit lesions, including fatty acid degradation and the citric acid cycle. The top two enriched pathways were fat degradation and oxidative phosphorylation (Figure 3D). In the metabolic process of lipid degradation, CPT1A was upregulated most significantly among the differentially expressed proteins in the SSR group (Figure 3E). In the gene set enrichment analysis (GSEA), fatty acid degradation was upregulated, with a normalized enrichment score of 1.78, while oxidative phosphorylation was downregulated, with an NES of −1.38 (Figure 3F). A protein–metabolism interaction network was developed, and the extracted LCACs and LCACs-related regulatory molecules were divided into subsets. Five hub molecules (excluding LCACs) were identified through the maximal clique centrality value in the subsets. The values were ranked, and CPT1A was the highest ranked metabolic enzyme to show an interaction with LCACs (Figure 3G). CPT1A expression in the infarcted myocardium was evaluated by Western blotting. The results indicated that compared with the control group, CPT1A expression was significantly increased in the SSR group (Figure S3B). Double immunofluorescence staining for CPT1A and CD31 (endothelial cells) (red) or cTnT (cardiomyocytes) (green) was performed to determine the cellular localization of CPT1A. CPT1A immunopositivity was significantly higher in the SSR group than in the control group, and immunofluorescence showed mainly colocalization of CPT1A with endothelial cells, but not cardiomyocytes (Figure 3H). In addition, enrichment of the GSEA pathways showed that revascularization of non-culprit lesions upregulated the hemodynamic shear stress pathway (Figure S3C). These findings indicate that endothelial CPT1A serves as the primary regulator of LCACs in response to hemodynamic shear stress and reperfusion injury, specifically in non-culprit lesions.

### LCAC metabolic dysregulation triggers cardiomyocyte death and mitochondrial dysfunction

**Figure 4:**
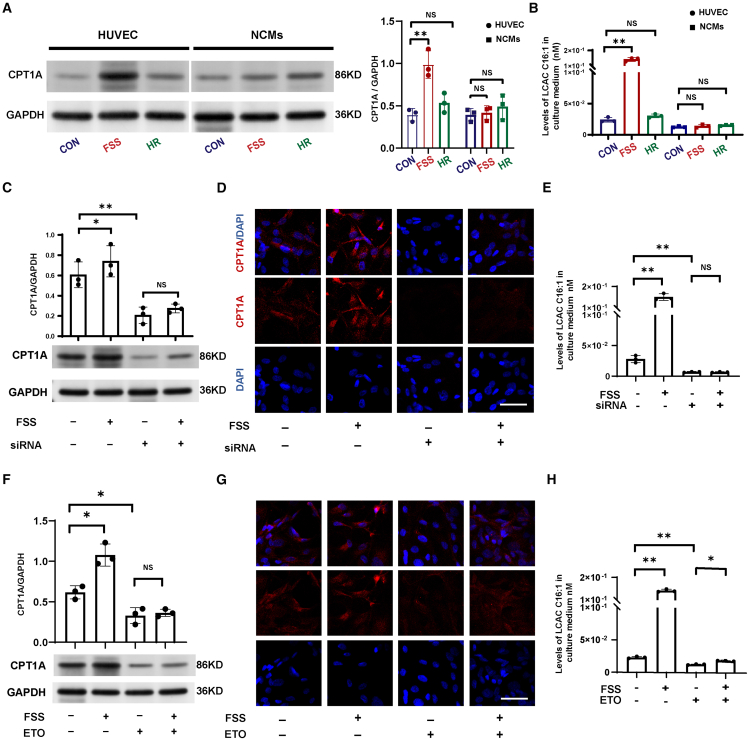
Shear stress-dependent endothelial CPT1A regulates LCAC metabolism *in vitro* (A) CPT1A protein expression was quantified in HUVECs and NCMs that were exposed to FSS or HR (*n* = 3/group). Data were presented as means ± standard deviations as indicated. ∗∗*p* < 0.01 in a Student’s unpaired *t* test. (B) Concentrations of LCAC C16:1 in culture medium of HUVECs and NCMs that were exposed to FSS or HR (*n* = 3/group). Data were presented as means ± standard deviations as indicated. ∗∗*p* < 0.01 in a Student’s unpaired *t* test. (C) Protein CPT1A expression quantitation treated with FSS/siRNA-CPT1A in HUVECs (*n* = 3/group). Data were presented as means ± standard deviations as indicated. ∗*p* < 0.05 and ∗∗*p* < 0.01 in a Student’s unpaired *t* test. (D) Representative fluorescence image of CPT1A treated with FSS/siRNA-CPT1A in HUVECs. Scale bar, 100 μm. (E) Concentrations of LCAC C16:1 in culture medium treated with FSS/siRNA-CPT1A in HUVECs (*n* = 3/group). Data were presented as means ± standard deviations as indicated. ∗∗*p* < 0.01 in a Student’s unpaired *t* test. (F) Protein CPT1A expression quantitation treated with FSS/ETO in HUVECs (*n* = 3/group). Data were presented as means ± standard deviations as indicated. ∗*p* < 0.05 in a Student’s unpaired *t* test. (G) Representative fluorescence image of CPT1A treated with FSS/ETO in HUVECs. Scale bar, 100 μm. (H) Concentrations of LCAC C16:1 in culture medium treated with FSS/ETO in HUVECs (*n* = 3/group). Data were presented as means ± standard deviations as indicated. ∗*p* < 0.05 and ∗∗*p* < 0.01 in a Student’s unpaired *t* test. HUVEC, human umbilical vein endothelial cell; NCMs, mouse neonatal cardiomyocytes; FSS, fluid shear stress; HR, hypoxia reoxygenation; CON, control; siRNA, siRNA-CPT1A; ETO, etomoxir; LCAC, long-chain acylcarnitine.

We conducted experiments to verify whether shear stress-dependent LCACs metabolic dysregulation caused cardiomyocyte death and mitochondrial dysfunction. The proteomics analysis showed that certain proteins (PECAM1, PPKAA1, MAPK14, RAC1, and RAC2) in the hemodynamic shear stress pathway were positively correlated with expression of CPT1A (*p* < 0.05) (Figure S3D). We measured CPT1A protein levels in endothelial cells under hypoxic (non-culprit) and anoxic (culprit) conditions. Both hypoxia and anoxia upregulated CPT1A expression in endothelial cells compared to the control group (*p* < 0.05). However, the increase in the anoxia group was not statistically greater than that in the hypoxia group (*p* = 0.0649), suggesting that hemodynamic shear stress during revascularization may play a more critical role in driving CPT1A upregulation in endothelial cells (Figures S4A and S4B). To determine the main effect of hemodynamic shear stress stimulation on CPT1A in endothelial cells, we exposed both human umbilical vein endothelial cells (HUVECs) and mouse neonatal cardiomyocytes (NCMs) to fluid shear stress (FSS) or hypoxia reoxygenation. The western blotting results for CPT1A expression suggested that the most significant increase in CPT1A expression occurred in HUVECs stimulated by shear force stress rather than hypoxia–reoxygenation. The increase in CPT1A expression in NCMs was not significant under shear force stress and hypoxia followed by 12-h reoxygenation (Figure 4A). However, CPT1A expression was upregulated in NCMs, with the increase continuing from 24 to 36 h reoxygenation (Figures S4D and S4E). This result suggests a significant increase in CPT1A levels in the cardiomyocytes during the late reoxygenation phase. The level of LCAC C16:1 produced by HUVECs in the culture medium under the stimulation of strong shear force increased significantly (Figure 4B). After siRNA interference of CPT1A expression in HUVECs, hemodynamic shear stress failed to induce an increase in CPT1A expression in HUVECs or an increase in the concentration of LCAC C16:1 in the culture medium (Figures 4C–4E). Similarly, ETO treatment also abolished CPT1A expression in HUVECs or LCACs in the culture medium induced by hemodynamic shear stress stimulation (Figures 4F–4H).

**Figure 5:**
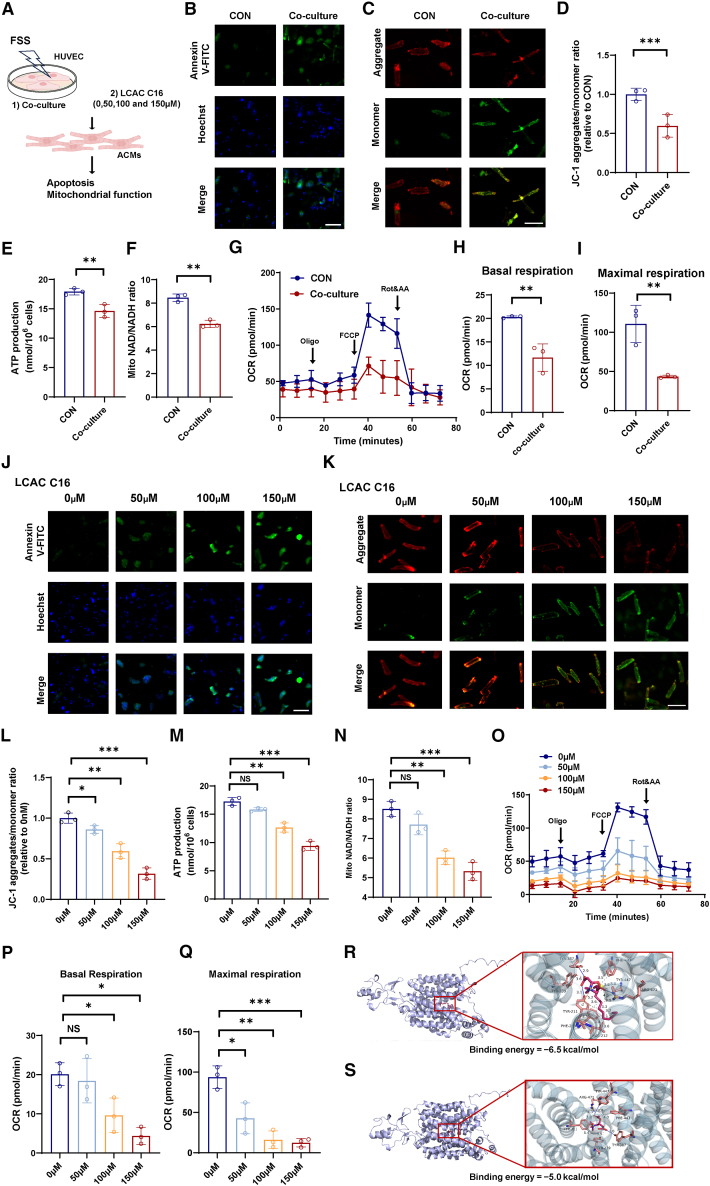
Endothelial cell-derived LCACs affect cardiomyocytes, leading to cell apoptosis and mitochondrial dysfunction *in vitro* (A) Schematic diagram of the *in vitro* experimental protocol: (1) HUVECs subjected to FSS stimulation were subsequently co-cultured with ACMs or (2) ACMs were treated with various concentrations of LCAC C16 (0, 50, 100, and 150 μM). (B) Apoptotic cells were labeled with annexin V-FITC (green fluorescence) in ACMs from the co-culture and control groups. Scale bar, 100 μm. (C) Representative photomicrographs of JC-1 staining in ACMs from the co-culture and control groups. Scale bar, 100 μm. (D) Quantification of the JC-1 fluorescence ratio in ACMs from the co-culture and control groups (*n* = 3/group). Data were presented as means ± standard deviations as indicated. ∗∗∗*p* < 0.001 in a Student’s unpaired *t* test. (E) ATP levels of ACMs from the co-culture and control groups (*n* = 3/group). Data were presented as means ± standard deviations as indicated. ∗∗*p* < 0.01 in a Student’s unpaired *t* test. (F) Mito NAD/NADH ratio of ACMs from the co-culture and control groups (*n* = 3/group). Data were presented as means ± standard deviations as indicated.∗∗*p* < 0.01 in a Student’s unpaired *t* test. (G–I) Oxygen consumption rate of ACMs from the co-culture and control groups using the seahorse system. The basal respiration and maximal respiration were assessed (*n* = 3/group). Data were presented as means ± standard deviations as indicated.∗∗*p* < 0.01 in a Student’s unpaired *t* test. (J) Apoptotic cells were labeled with annexin V-FITC (green fluorescence) in ACMs treated with various concentrations of LCAC C16 (0, 50, 100, and 150 μM). Scale bar, 100 μm. (K) Representative photomicrographs of JC-1 staining in ACMs treated with various concentrations of LCAC C16 (0, 50, 100, and 150 μM). Scale bar, 100 μm. (L) Quantification of the JC-1 fluorescence ratio in ACMs treated with various concentrations of LCAC C16 (0, 50, 100, and 150 μM) (*n* = 3/group). Data were presented as means ± standard deviations as indicated. ∗*p* < 0.05, ∗∗*p* < 0.01, and ∗∗∗*p* < 0.001 in a Student’s unpaired *t* test. (M) ATP levels of ACMs treated with various concentrations of LCAC C16 (0, 50, 100, and 150 μM) (*n* = 3/group). Data were presented as means ± standard deviations as indicated. ∗∗*p* < 0.01 and ∗∗∗*p* < 0.001 in a Student’s unpaired *t* test. (N) Mito NAD/NADH ratio of ACMs treated with various concentrations of LCAC C16 (0, 50, 100, and 150 μM) (*n* = 3/group). Data were presented as means ± standard deviations as indicated. ∗∗*p* < 0.01 and ∗∗∗*p* < 0.001 in a Student’s unpaired *t* test. (O–Q) Oxygen consumption rate of ACMs treated with various concentrations of LCAC C16 (0, 50, 100, and 150 μM) using the Seahorse system. The basal respiration and maximal respiration were assessed (*n* = 3/group). Data were presented as means ± standard deviations as indicated. ∗*p* < 0.05, ∗∗*p* < 0.01, and ∗∗∗*p* < 0.001 in a Student’s unpaired *t* test. (R and S) Molecular docking results of OCTN2 and palmitoylcarnitine (binding energy = −6.5 kcal/mol) and acetyl carnitine (binding energy = −5.0 kcal/mol). HUVEC, human umbilical vein endothelial cell; ACMs, adult rat cardiomyocytes; FSS, fluid shear stress; CON, control; OCR, oxygen consumption rate; LCAC, long-chain acylcarnitine; OCTN2, type 2 organic cation transporter.

After simultaneous reperfusion, upregulation of CPT1A in endothelial cells led to an increase in the concentration of circulating LCACs. To investigate how the excess LCACs affected cardiomyocyte function, adult rat cardiomyocytes (ACMs) were co-cultured with HUVECs stimulated by blood flow shear force or treated with different concentration gradients of LCAC C16:1 (Figure 5A). After 18 h of co-culture, the morphology of ACMs changed from rod-shaped to spherical (Figure S5A). Annexin V-fluorescein isothiocyanate (FITC) green fluorescent staining demonstrated that co-culture induced mitochondrial dysfunction and cell apoptosis (Figure 5B). JC-1 staining showed that the ratio of JC-1 aggregates to JC-1 monomers decreased, representing the lower mitochondrial membrane potential under co-culture conditions (Figures 5C and 5D). In addition, co-culture decreased ATP production and mitochondrial NAD^+^/NAD hydrogen (NADH) ratio in ACMs (Figures 5E and 5F).

Next, we assessed the effects of co-culture on mitochondrial respiratory capacity by measuring the oxygen consumption rate (OCR), a key indicator of mitochondrial function in ACMs. Co-culture led to decreases in basal respiration and maximal respiration, as indicated by the OCR (Figures 5G–5I). As the LCAC concentration increased, we observed an intensification of cell apoptosis and a decrease in cell viability, particularly at concentrations of 100 and 150 μM (Figures 5J and S5B). JC-1 staining showed that as the concentration of LCAC increased, the ratio of JC-1 aggregates to JC-1 monomers decreased (Figures 5K and 5L). Additionally, ATP production and the mitochondrial NAD^+^/NADH ratio in ACMs decreased with an increase in LCAC stimulation, especially at concentrations greater than 50 μM (Figures 5M and 5N). Treatment with a high LCAC concentration resulted in reduced basal and maximal respiration, as indicated by the OCR (Figures 5O–5Q). Similar results were obtained by stimulating NCMs under the same conditions (Figure S6). Carnitine has been shown to penetrate cells and exert its effects via OCTN2.^21^ To explore whether LCACs utilize a similar pathway to enter cells and exert their effects, we performed molecular docking of palmitoylcarnitine with OCTN2. The results revealed a minimum binding energy of −6.5 kcal/mol for the interaction between palmitoylcarnitine and OCTN2, indicating a strong binding affinity compared to the minimum binding energy of acetyl-L-carnitine and OCTN2 (Figures 5R and 5S). High LCAC concentrations (150 μM) upregulated OCTN2 protein expression; this effect was significantly attenuated by treatment with the OCTN2 inhibitor, MET-88 (Figures S7A and S7B). Furthermore, treatment with 150 μM LCACs significantly increased their intracellular accumulation. Inhibition of OCTN2 with MET-88 significantly reduced this accumulation (Figure S7C). Finally, we found that OCTN2 inhibition attenuated the LCAC-induced loss of mitochondrial membrane potential, as measured by JC-1 staining (Figure S7D). The above results indicate that LCACs enters cardiomyocytes through OCTN2, leading to mitochondrial dysfunction(Figure S7E).

### LCAC C16 suppressed mitochondrial biogenesis by inhibiting Ppargc1a expression

**Figure 6:**
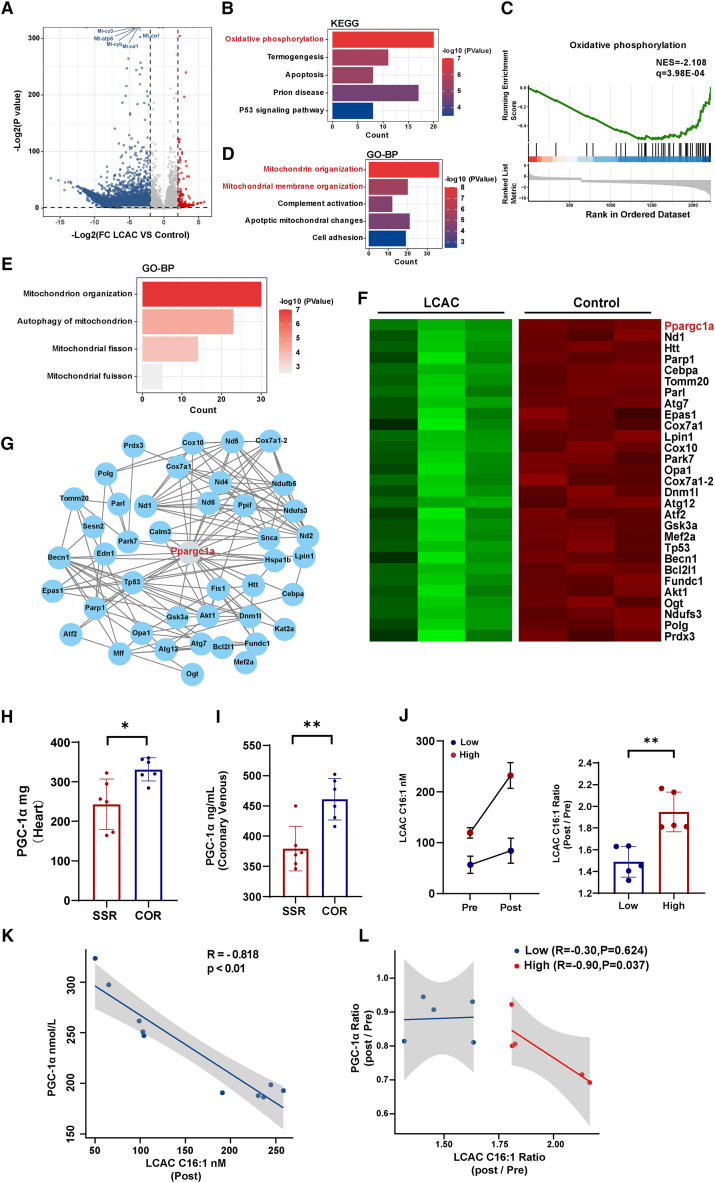
LCAC C16 causes cardiac mitochondrial homeostasis disruption by inhibiting the expression of PPARGC1A (A)Transcriptome analysis volcano plot of mRNA expression ACMs treated with LCAC C16. (B) KEGG pathway enrichment results of differentially expressed genes of ACMs treated with LCAC C16. (C) GSEA of oxidative phosphorylation pathways. (D) GO biological process enrichment in LCAC C16-treated ACMs. (E and F) Gene Ontology analysis of MQC system-related biology process changes and heatmap of key genes in the mitochondrion organization from RNA sequencing analysis. (G) MQC system-related differentially expressed genes interaction network. (H and I) Expression of PGC-1α, an MQC system-related biomarker, in myocardial tissue and coronary venous blood from SSR and COR groups in a swine model of AMI (*n* = 6/group). Data were presented as means ± standard deviations as indicated. ∗*p* < 0.05 and ∗∗*p* < 0.01 in a Student’s unpaired *t* test. (J) Comparison of preoperative and postoperative levels of LCAC C16:1 between high- and low-concentration groups in patients with AMI and multivessel disease (*n* = 5/group). Data were presented as means ± standard deviations as indicated. ∗∗*p* < 0.01 in a Student’s unpaired *t* test. (K) Correlation between postoperative serum LCAC C16:1 levels and PGC-1α expression in patients with AMI and multivessel disease (*n* = 10). R, correlation coefficient; Spearman correlation analysis. (L) Correlation between ratio of LCAC C16:1 levels and ratio of PGC-1α (Post/Pre) in high and low LCAC C16:1 concentration groups (*n* = 5/group).R, correlation coefficient; Spearman correlation analysis. R, correlation coefficient; Spearman correlation analysis. FC, fold change; KEGG, Kyoto Encyclopedia of Genes and Genomes; LCAC, long-chain acylcarnitine; GO-BP, Gene Ontology biological process; ACMs, adult rat cardiomyocytes; GSEA, Gene Set Enrichment Analysis; NES, normalized enrichment score; MQC, mitochondrial quality control; SSR, single-stage revascularization group; COR, culprit-only revascularization group; AMI, acute myocardial infarction; Pre, preoperative; Post, postoperative.

To determine how LCACs affect mitochondrial dysfunction in cardiomyocytes, we investigated the transcriptomic differences between ACMs stimulated with a high LCAC C16 concentration (150 μM) and control cells by transcriptome sequencing. Transcriptomics analysis showed downregulation of mitochondria-related genes, including Mt-co1, Mt-cyb, and Mt-nd1, among others, in the high-concentration LCAC group (Figure 6A). We assessed the impact of high LCAC concentration on cardiomyocytes using differential gene expression analysis, GSEA, KEGG enrichment analysis, and Gene Ontology analysis. The results showed that stimulation with a high LCAC concentration led to the inhibition of oxidative phosphorylation, which is consistent with the proteomics results from the large animal model (Figures 6B and 6C). Biological process analysis of differential gene expression revealed biological process terms associated with the mitochondria, such as mitochondrion organization and mitochondrial membrane organization (Figure 6D). Gene ontology analysis of differentially expressed mitochondria-related genes demonstrated that LCACs severely impair processes related to mitochondrial quality control. These processes—including mitochondrion organization, mitophagy, and mitochondrial fission—were disrupted, accompanied by the downregulation of mitochondrial quality control system genes in the LCAC group. (Figures 6E and 6F). Gene-gene interaction network analysis of mitochondrial quality control-related differentially expressed genes identified *Ppargc1a* as a key regulator of mitochondrial biogenesis (Figure 6G).

In the porcine model, myocardial and coronary vein levels of PGC-1α (*PPARGC1A*), a key biomarker of the mitochondrial quality control system, were downregulated in the SSR group compared to the control group (Figures 6H and 6I). Five patients had blood samples collected for LCAC testing before and 24 h after surgery. The median concentration of LCAC C16:1 was used as the cutoff to divide the concentration into high (>90 nM) and low (<90 nM) groups. The results showed that patients with a high LCAC C16:1concentration before surgery had a more significant increase in LCAC C16:1 after surgery (Figure 6J). Furthermore, there was a negative correlation between postoperative circulating LCAC C16:1 and PGC-1α (Figure 6K). In patients with high preoperative LCAC C16:1 level (>90 nM), there was a significant negative correlation between concentrations of postoperative PGC-1α and LCAC C16:1 (Figure 6L).

### Modulation of CPT1A-LCAC pathway alters post-ischemic cardiac remodeling and cardiac injury

**Figure 7:**
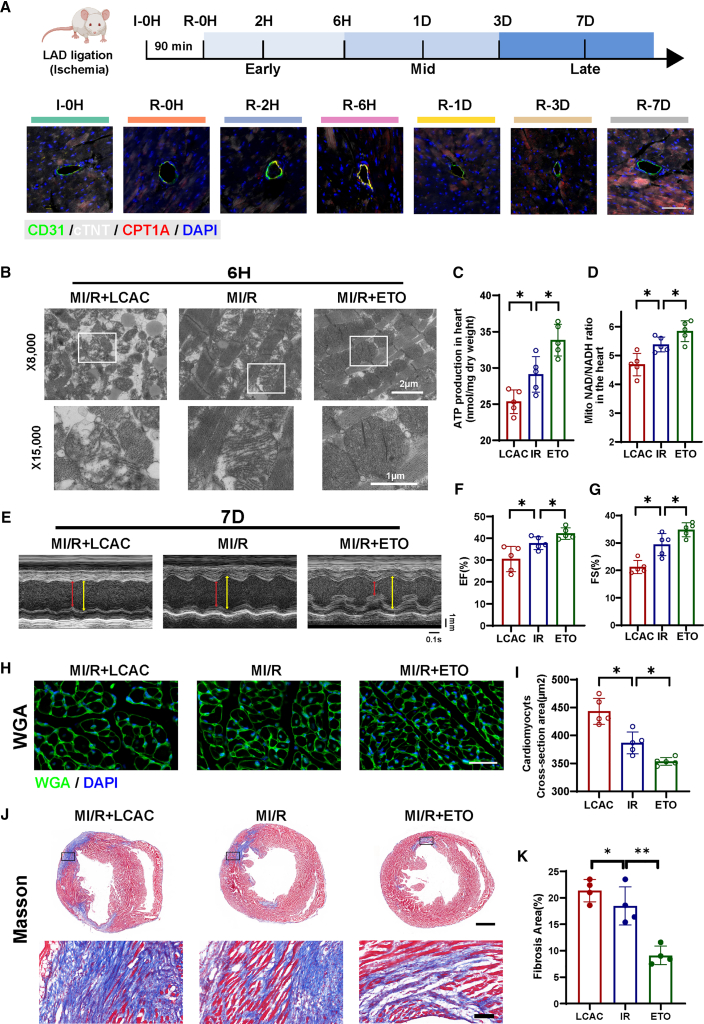
Modulation of CPT1A-LCAC pathway alters post-ischemic cardiac remodeling and cardiac injury (A) Myocardial ischemia-reperfusion injury was induced by ligation of the left anterior descending coronary artery in the middle of the mid-ant region (I-0H) for 90 min and then reperfusion for the indicated time points (R-0H, 2H, 6H, 1D, 3D, and 7D). Cardiac tissues were processed for immunofluorescence staining. Immunostaining of CD31, cTNT, and CPT1A (red) in infarcted hearts. Scale bar: 50 μm. (B) Transmission electron microscopy images (×8,000 and ×15,000) of representative mitochondrial areas in infarcted hearts from the LCAC, IR, and ETO groups. (C) ATP production in infarcted hearts from the LCAC, IR, and ETO groups (*n* = 5/group). Data were presented as means ± standard deviations as indicated. ∗*p* < 0.05 in a Student’s unpaired *t* test. (D) Mito NAD/NADH ratio in infarcted hearts from the LCAC, IR, and ETO groups (*n* = 5/group). Data were presented as means ± standard deviations as indicated. ∗*p* < 0.05 in a Student’s unpaired *t* test. (E) Representative M-mode echocardiographic images of LCAC, IR, and ETO groups. Scale bar in mm/s on the right, and time stamp in seconds at the bottom. (F and G) Echocardiographic quantifications of LCAC, IR, and ETO groups (*n* = 5/group). Shown in the statistical graph are left ventricular ejection fraction (EF) and left ventricular fractional shortening (FS). Data were presented as means ± standard deviations as indicated. ∗*p* < 0.05 in a Student’s unpaired *t* test. (H and I) Heart tissue WGA staining to quantification of cross-sectional area of cardiomyocytes in LCAC, IR, and ETO groups (*n* = 5/group). Scale bar: 25 μm. Data were presented as means ± standard deviations as indicated. ∗*p* < 0.05 in a Student’s unpaired *t* test. (J and K) Heart tissue Masson trichrome staining of LCAC, IR, and ETO groups (scale bar: 2 and 25 μm). Quantification of cardiac fibrosis area from Masson trichrome-stained sections in LCAC, IR, and ETO groups (*n* = 4/group). Data were presented as means ± standard deviations as indicated. ∗*p* < 0.05 and ∗∗*p* < 0.01 in a Student’s unpaired *t* test. LAD, left anterior descending coronary artery; MI/R and IR, myocardial ischemia-reperfusion injury; LCAC, long-chain acylcarnitine; ETO, etomoxir; EF, ejection fraction; FS, fractional shortening; WGA, wheat germ agglutinin staining.

To determine the role of the CPT1A-LCAC pathway in post-ischemic cardiac injury, we subjected mice to MI/R injury by ligating the left anterior descending coronary artery at its midportion for 90 min. Mice were sacrificed at various time points after reperfusion, and heart tissue was collected for immunofluorescence analysis. Three-color immunofluorescence to analyze the distribution of CPT1A expression in the heart before ischemic injury (I-0H) and at early (R-0H, R-2H, and R-6H), middle (R-1D), and late (R-3D and R-7D) stages after reperfusion. We observed robust expression of CPT1A in endothelial cells, as indicated by its colocalization with CD31 in the early stages after reperfusion (R-6H). However, after 7 days of reperfusion (R-7D), CPT1A was more frequently co-localized with cTNT (Figure 7A). During the ischemic phase in fasted mice, we administered LCAC and ETO via intraperitoneal injection, followed by reperfusion injury for 6 h (R-6H) or 7 days (R-7D). After intraperitoneal injection for 6 h, three groups of mouse serum and heart tissue were obtained for LCAC C16:1 detection. The results showed that after injection of LCAC, the LCAC levels in the heart and serum increased, while the ETO treatment group reduced their accumulation in the heart and serum (Figure S8). Pathology examinations, and molecular biology analyses to investigate the effects of LCAC metabolic dysregulation. Transmission electron microscopy was used to examine mitochondrial morphology and cardiac muscle structure across three treatment groups. We found that LCAC supplementation led to intermyofibrillar swelling, disarrayed myofilaments, and mitochondrial structural changes, including mitochondrial swelling, loss of cristae, and vacuolization. In contrast, treatment with ETO improved myocardial structure and mitochondrial morphology following ischemia-reperfusion injury (R-6H) (Figure 7B). Cardiac ATP biosynthesis and NAD+ production was decreased in mice supplemented with LCAC. In contrast, the ETO group showed the opposite trend compared to WT mice following MI/R injury (R-6H) (Figures 7C and 7D). Echocardiography revealed that left ventricular ejection fraction and left ventricular fractional shortening were worse in the LCAC group after 7 days of reperfusion injury. However, the ETO group showed opposite results, indicating improved cardiac function (Figures 7E–7G). Representative Wheat germ agglutinin-stained images and myocyte cross-sectional area show that LCAC supplementation exacerbates cardiac hypertrophy 7 days after reperfusion injury, whereas ETO treatment reduces myocardial hypertrophy (Figures 7H and 7I). Masson staining illustrated that LCAC supplementation facilitated cardiac fibrosis, whereas ETO treatment eliminated the cardiac fibrosis (Figures 7J and 7K).

## Discussion

We conducted a multi-omics analysis to identify metabolic markers for distinguishing high-risk patients and potential targets for treating MI/R injury in non-culprit lesions. We found that patients with a high circulating LCAC C16:1 concentration (>90 nM) had a poor prognosis after complete revascularization. The combination of LCAC C16:1 level with SYNTAX or GRACE scores demonstrated strong predictive capabilities, effectively discriminating between individuals at high risk and those at low risk. Moreover, we developed a swine model of AMI with multivessel disease to mimic reperfusion injury in non-culprit lesions and found that simultaneous revascularization of non-culprit lesions led to a greater degree of cardiac injury, which manifested as mitochondrial dysfunction and apoptosis in the myocardium. We also discovered that the activation of CPT1A in endothelial cells, driven by FSS induced during recanalization of non-culprit lesions, was the primary determinant for LCAC release. The released LCACs entered cardiomyocytes and downregulated Ppargc1a expression, which is a master regulator of mitochondrial biogenesis and energy metabolism, resulting in the inhibition of mitochondrial biogenesis.

### Risks of complete revascularization in multivessel disease

PCI is an important treatment for patients with AMI. If the non-culprit lesions are not opened, the recurrence rate of myocardial infarction may increase.^22^^,^^23^ Randomized controlled trials have provided compelling evidence that performing multivessel PCI for complete revascularization is more effective than opting for PCI focused solely on the culprit lesion. This comprehensive approach significantly reduces the risk of cardiovascular death, myocardial infarction, and the need for ischemia-driven revascularization within the first year.^24^^,^^25^ Therefore, when AMI with multivessel disease occurs, non-culprit lesions should be treated as soon as possible to reduce the risk of recurrence of non-culprit-derived culprit lesions.^3^ The increase in blood flow to culprit lesions during simultaneous revascularization can reduce infarct size to a certain extent and reduce the incidence of adverse events related to non-culprit lesions.^8^^,^^26^ A previous observational study, CvLPRIT, showed that simultaneous revascularization was associated with long-term improvement of left ventricular systolic function after surgery compared with staged revascularization.^27^ Nonetheless, it is important to note that simultaneous revascularization may not be the optimal choice for every patient, as not all individuals stand to benefit from this intervention when considering the associated risks. Studies have demonstrated that in certain cases, the occurrence of heart failure and cardiac-related mortality following PCI is significantly greater than that observed with multi-stage revascularization.^28^^,^^29^ Unfortunately, in clinical practice, identifying key molecules underlying acute cardiac injury due to non-infarction-related recanalization and identifying high-risk patients is extremely difficult.^30^

### Role of hemodynamic changes and endothelial-cardiomyocyte crosstalk in MI/R injury

MI/R has become a major factor leading to poor outcomes after PCI in patients with AMI. The restoration of coronary artery blood flow, while essential, inevitably leads to changes in shear stress. FSS, which is generated by the directional shearing of blood flow, exerts direct effects on endothelial cells.^31^ Endothelial cells in non-culprit lesions experience continuous exposure to low-intensity flow shear forces. Endothelial cells in non-culprit lesions, exposed to sudden changes in pressure and flow, can respond with vasoconstriction or spasm due to impaired endothelial-mediated dilation. This phenomenon further contributed to the instability of non-culprit lesions.^32^ Abrupt elevations in these low-intensity shear forces following complete revascularization might lead to increased endothelial cell membrane permeability, membrane phospholipid degradation, and cell membrane peroxidation, further affecting lipid metabolism disorders.^33^ The interaction between endothelial cells and cardiomyocytes during MI/R injury plays a critical role in the extent of tissue damage and recovery.^34^ Endothelial cells and cardiomyocytes communicate through paracrine signaling, with endothelial cells releasing calcium and nitric oxide, among other factors, which either promote cell survival or aggravate cardiomyocyte injury.^35^ In this study, using a porcine model of AMI with multivessel disease, we found that on the basis of culprit lesion recanalization, early recanalization of non-culprit lesions led to an increase in flow shear force, elevated CPT1A activity, and a further increase in circulating LCACs. Similar findings were also observed in mouse models and *in vitro*. Therefore, LCACs released by the endothelium may participate in endothelial–cardiomyocyte crosstalk. At high LCAC concentrations, LCACs can enter cardiomyocytes via OCTN1, accumulate within the cells, and induce mitochondrial dysfunction, ultimately leading to myocardial remodeling.

### Molecular mechanism of LCAC-induced mitochondrial dysfunction

LCACs are intermediate products of fatty acid oxidation, and their production involves fatty acid metabolism and acylcarnitine transport.^36^ The molecular mechanism by which LCACs induce mitochondrial dysfunction in the myocardium remains unclear.^37^ Our study revealed that LCACs enter cardiomyocytes via OCTN1, thereby inhibiting Ppargc1a and suppressing mitochondrial biogenesis, which ultimately leads to cardiac mitochondrial dysfunction. PGC-1α is a key regulator of mitochondrial biogenesis and activates the transcription of genes involved in mitochondrial DNA replication, oxidative phosphorylation, and energy metabolism, playing a crucial role in enhancing mitochondrial function and adaptation to physiological stress.^38^ This result provides further insights into the molecular mechanism of non-culprit lesion MI/R injury caused by LCACs.

### Preoperative LCACs levels and targeting CPT1A as an intervention

In the present study, a high preoperative concentration of circulating plasma LCAC C16:1 (>90 nM) was independently associated with adverse cardiovascular events following immediate multivessel PCI. At the same time, the cumulative impact of elevated preoperative LCAC on postoperative cascade amplification was more pronounced compared with LCAC at a lower concentration. This is primarily because the preoperative concentration of LCAC partly reflects CPT1A activity, with a higher LCAC concentration indicating greater CPT1A activity. Under the influence of flow shear stress, the activity of CPT1A in endothelial cells was further amplified, leading to increased release of circulating LCACs. Therefore, we targeted CPT1A as a point of intervention with inhibitors. The results showed that following the use of ETO, the stimulatory effect of FSS on endothelial cells had significantly diminished. Concurrently, the concentration of LCAC released into the culture medium had also decreased. Previous research has suggested that ETO could serve as a potential treatment for heart failure by inhibiting fatty acid oxidation, alleviating the cardiac burden and reducing the energy demands of myocardial cells.^39^ Despite showing promise in animal models and *in vitro* experiments, the efficacy of ETO has not been fully confirmed in clinical trials. Long-term use of ETO could also be associated with some side effects and safety concerns, such as hepatotoxicity.^40^ Therefore, short-term use of ETO might be an effective strategy for treating MI/R injury of non-culprit lesions. Small molecule inhibitors of CPT1A or OCTN2 warrant further exploration for translational implications and future directions.

### Limitations of the study

The pathological mechanisms underlying disrupted LCAC metabolism in endothelial cells, directly stimulated by hemodynamic injury post-reperfusion, remain unclear. Further comprehensive research is required to elucidate the induction of mitochondrial dysfunction and proliferation disorders in cardiomyocytes by LCACs. In addition, blood samples were collected from the peripheral veins of the patients, which is inconsistent with the coronary vein blood samples obtained in the swine model.

### Conclusions

In summary, accumulation of endothelial cell-derived long-chain acylcarnitine result in decline of mitochondrial quality control, aggravating ischemia-reperfusion injury of non-culprit lesions. High-risk patients can be identified by preoperatively detecting LCACs in the circulation, and the CPT1A-LCAC pathway represents a promising therapeutic target for this patient population.

## Resource availability

### Lead contact

Further information and requests for resources and reagents should be directed to and will be fulfilled by the lead contact, Yuan Wang (wangyuan980510@163.com).

### Materials availability

This study did not generate new reagents or materials.

### Data and code availability

• Raw bulk RNA sequencing data have been deposited in the Gene Expression Omnibus. Metabolomics data have been deposited in MetaboLights. Accession numbers are listed in the key resources table.
• This paper does not report original code.
• Any additional information required to reanalyze the data reported in this work paper is available from the lead contact upon request.

## Acknowledgments

This study was supported by the 10.13039/501100012166National Key R&D Program of China (grant no. 2021YFA0805100), National Natural Science Foundation of China Major Program (grant no. 91939303), 10.13039/501100001809National Science Foundation of China (82530014), and the 10.13039/501100005088Beijing Municipal Health Commission (grant no. 11000025T000003321782). The authors appreciate all the sites that contributed to recruitment, the patients and their families who participated in this study, and the participating study teams. We thank Tingting Hou and Yingna Guo from Peking University for their assistance with the isolation of adult rat cardiomyocytes.

## Author contributions

Conceptualization, R.L., S.Y., J.D., and Y.W.; methodology, R.L., Y.L., S.Y., H.G., F.L., Xue Wang, X.T., L.W., J.Q., and Xiujie Wang; formal analysis, R.L., L.R., Xiujie Wang, and J.Q.; investigation, R.L., Y.L., Z.W., F.L., and W.Y.; resources, R.L., Y.L., S.Y., W.C., F.L., W.Y., J.D., and Y.W.; data curation, R.L., Y.L., and Y.W.; writing – original draft, R.L. and Y.W.; writing – review and editing, all authors.

## Declaration of interests

The authors declare no competing interests.

## STAR★Methods

### Key resources table

**Table:** 

REAGENT or RESOURCE	SOURCE	IDENTIFIER
**Antibodies**		

CD31 antibody	Abcam	Cat# ab281583; RRID:AB_3096925
OCTN2 antibody	Proteintech	Cat# 16331-1-AP; RRID:AB_2191406
CPT1A antibody	Abcam	Cat# ab234111; RRID:AB_2864319
CTNT antibody	Proteintech	Cat# 15513-1-AP; RRID:AB_2206563

**Biological samples**		

Human blood sample	This paper	N/A
Pig blood sample	This paper	N/A
Pig heart tissue	This paper	N/A
Mouse blood sample	This paper	N/A
Mouse heart tissue	This paper	N/A

**Chemicals, peptides, and recombinant proteins**		

L-Palmitoylcarnitine chloride	MedChemExpress	Cat# HY-113147A
Meldonium	MedChemExpress	Cat# HY-B1836
Etomoxir	MedChemExpress	Cat# HY-50202
Palmitoyl-L-carnitine	Sigma Aldrich	Cat# P1645
Wheat germ agglutinin	Sigma Aldrich	Cat# L4859

**Critical commercial assays**		

PGC-1α assay kit	Fankew	Cat# F11500-B, Cat# F0054-PB
Pig Cardiac Troponin-I assay kit	Kamiya Biomedical	Cat# KT-474
Malondialdehyde content assay Kit	Solarbio	Cat# BC0025
JC-1 assay Kit	Solarbio	Cat# M8650
ATP assay kit	Beyotime	Cat# S0027
NAD+/NADH assay kit	Beyotime	Cat# S0176S
Mitochondrial Membrane Potential Detection Kit	Beyotime	Cat# C1071
CCK8 assay	Beyotime	Cat# C0037
Seahorse XF Cell ito Stress Test kit	Agilent	Cat# 103015-100
MxP® Quant 500 targeted metabolomics kit	Biocrates Life Sciences AG	https://biocrates.com/mxp-quant-500-kit/
ACQUITY 2D UPLC	Waters	https://www.waters.com/nextgen/us/en.html
Q Exactive (QE) hybrid Quadrupole-Orbitrap mass spectrometer	Thermo Scientific	https://www.thermofisher.cn/
Sciex Triple Quad 6500	Sciex	https://sciex.com/
Q Exactive HF mass spectrometer	Thermo Scientific	https://www.thermofisher.cn/
Easy-nLC 1000 nanoflow LC system	Thermo Scientific	https://www.thermofisher.cn/
Illumina NovaSeq 6000	Illumina	https://emea.illumina.com/systems/sequencing-platforms/novaseq.html

**Deposited data**		

Raw bulk RNA-seq data	GEO	GEO: GSE308471
Metabolomics data	MetaboLights	MTBLS6651

**Experimental models: Cell lines**		

HUVEC	ScienCell	Cat# 8000
NCM	This paper	N/A
ACM	This paper	N/A

**Experimental models: Organisms/strains**		

C57BL/6J Mice	This paper	N/A
Guangxi Bama miniature pigs	This paper	N/A

**Oligonucleotides**		

See Table S5 for oligonucleotide sequences	This paper	N/A

**Software and algorithms**		

ImageJ	NIH	https://imagej.nih.gov/ij/
R version 4.2.2	R-Project	https://cran.r-project.org/
Cytoscpae 3.9.1	Cytoscape Consortium	https://cytoscape.org/
GraphPad Prism 8.3.0	GraphPad	https://www.graphpad.com/scientificsoftware/prism/
CaseViewer v2.2.1	3DHISTECH	https://www.3dhistech.com/caseviewer
GSEA v3.0	Subramanian et al.^41^	http://software.broadinstitute.org/gsea
SPSS 22.0	IBM	https://www.ibm.com/
AutoDock Vina	Trott and Olson^42^	https://github.com/ccsb-scripps/AutoDock-Vina

### Experimental model and study participant details

#### Study participants

Overall, 416 patients with AMI combined with a non-infarcted artery who underwent complete revascularization via PCI between February 2019 and March 2023 were retrospectively selected from multiple centers, including three hospitals (Beijing Anzhen Hospital, *n* = 350; Beijing Luhe Hospital, *n* = 23; The First Hospital of Shanxi Medical University, *n* = 43). The population for the multicenter cohort study was randomly divided into screening dataset, derivation cohort, and validation cohort.(i) The screening cohort (*n* = 39) was used to identify candidate metabolites relevant to myocardial injury after complete revascularization.(ii) The derivation cohort (*n* = 216) was used to evaluate the prognostic value of candidate metabolites for MACEs using an independent cohort from Anzhen Hospital.(iii) The validation cohort (*n* = 161) was used to verify the predictive performance of the metabolites in a multicenter cohort.

The clinical inclusion criteria included the following: age over 18 years, symptoms of chest pain, evidence of ischemic changes on an electrocardiogram or elevated myocardial enzyme levels, coronary angiography confirming infarct-related vessels along with at least two non-infarct-related vessels exhibiting stenosis of ≥70% as determined by digital subtraction angiography, and complete revascularization via PCI performed after admission. Troponin concentrations were measured during the perioperative period. The exclusion criteria were as follows: a single coronary artery or ischemic lesion <70%, bypass surgery, conservative treatment, aortic dissection, pulmonary embolism, a malignant tumor, autoimmune disease, serious infectious disease, trauma, a recent operation, severe heart failure with a left ventricular ejection fraction <20%, liver insufficiency (alanine aminotransferase concentrations >135 μm/L), severe renal insufficiency (creatinine concentrations >3.0 mg/dL), and blood borne infectious diseases, such as human immunodeficiency virus acquired immunodeficiency syndrome, hepatitis B, and hepatitis C.

After the initial hospitalization, the patients were followed up for 44.1 months (interquartile range 22.0–47.8 months). Periprocedural myocardial injury was defined as a cardiac troponin I (cTnI) increase of >50% above the highest preprocedural value in at least one of the postprocedural samples.^43^ MACEs were defined as all-cause death and rehospitalization for heart failure.^44^ The clinical characteristics of the 416 patients are summarized in Table S4. We assessed the potential association of sex with our primary outcomes. The results were consistent across male and female subgroups, suggesting that the observed effects are independent of sex.This study was conducted in accordance with the principles outlined in the Declaration of Helsinki. This study was approved by the Beijing Anzhen Hospital Ethics Review Board, and all patients provided written informed consent.

#### Porcine model

Twelve male Guangxi Bama miniature pigs (Tianjin Bainong Experimental Animal Breeding Technology Co. Ltd., China) aged 6 months (weighing 20 ± 3 kg) were used to develop the large animal model of AMI with multivessel disease, mimicking reperfusion injury in non-culprit lesions (Figure 2A). Fasted minipigs were restrained individually on a surgical bed. Anesthesia was induced via intravenous bolus injection of propofol (15 mg/kg; 10 mg/mL concentration, GuangDong JiaBo Pharmaceutical Co., LTD, China) administered through the marginal ear vein. Anesthesia was maintained with a continuous intravenous infusion of propofol at a rate of 4–6 mg/kg/h (equivalent to 20 mL/h for a 40–50 kg minipig). Depth of anesthesia was assessed clinically by cessation of spontaneous movement, fixed lateral eye position, and loss of muscular tone. Following induction, minipigs were intubated and mechanically ventilated with a 1:1 air-oxygen mixture (FiO_2_ 50%) at a flow rate of 3 L/min^45^ A filled balloon (6–8 atm) was used for ischemic preconditioning, and 50 mg lidocaine was injected intravenously (0.03 mg/kg/min) to reduce the occurrence of ventricular arrhythmia. A balloon matching the diameter of the distal end of the left anterior descending branch was inserted into the opening of the second diagonal branch of the left anterior descending artery (LAD). The balloon was expanded to 2.5–3.0 mm to represent an infarcted artery, completely occluding the blood vessel. Another balloon matching the diameter of the left circumflex artery (LCX). was inserted into the left circumflex artery. The balloon was inflated to 1.5–2.0 mm to represent the non-culprit lesions, creating a narrow occluded area of approximately 70% by digital subtraction angiography.^46^^,^^47^

After successfully establishing the large animal model, after 30 min, the animals were randomly divided into two groups for treatment. In the single-stage revascularization (SSR) group, the two balloon catheters were withdrawn simultaneously (*n* = 6) to model simultaneous recanalization of non-culprit lesions. In the culprit-only revascularization (COR) group (*n* = 6), which served as the control group, only the balloon in the left anterior descending artery was withdrawn. The two groups of animals were anesthetized to collect coronary cardiac vein sample at different time points and then euthanized to collect cardiac tissue specimens 90 min after the balloons were withdrawn.

For euthanasia, 10 mL super-saturated potassium chloride (25%) was injected intravenously in deep anesthesia. Plasma was obtained from the blood samples via centrifugation for 15 min at 1300 *g* at a temperature of 4°C, and the plasma was utilized for both molecular biology experiments and metabolomics analyses. Cardiac tissue was collected following perfusion with ice-cold phosphate-buffered saline. A proportion of the cardiac tissue was frozen in liquid nitrogen for use in subsequent molecular biology experiments, metabolomics analyses, and proteomics analyses, while the rest of the cardiac tissue was fixed in 4% formaldehyde for immunofluorescence staining.

The experimental protocol was approved by the Animal Care Committee of Capital Medical University, and the study was performed in compliance with the Animal Management Rules of the Chinese Ministry of Health (Document No. 55, 2001). All procedures conform to the guidelines from Directive 2010/63/EU of the European Parliament on the protection of animals used for scientific purposes or the NIH Guide for the Care and Use of Laboratory Animals.

#### Mouse model

The mouse ischemia-reperfusion model was established through transient ligation of the left anterior descending (LAD) coronary artery. Briefly, after an overnight fast, mice were anesthetized with an intraperitoneal injection of ketamine (100 mg/kg) and xylazine (10 mg/kg), and then intubated and shaved. The chest was then opened between the fourth and fifth intercostal spaces and stabilized with a chest expander. A 7-0 prolene suture was used to ligate the LAD, with a 2 mm-long 4-0 prolene suture placed at the ligation site as a cushion thread. Successful ligation was indicated by whitening of the ischemic area and elevation of ST-segments on the electrocardiogram (ECG). Afterward, the chest cavity was closed, and the mice were extubated and placed on a heating pad. Following a 90-min ischemic period, the chest was reopened, and the ligation was released to allow reperfusion, indicated by the reddening of the previously pale ischemic area. Finally, the chest was closed, and the mice were allowed to recover. Ventilator parameters for this procedure were set as follows: respiration ratio 1:1, frequency 90–100 breaths per minute, and tidal volume 1–2 mL. Mice were euthanized (i.p 120 mg/kg ketamine, 12 mg/kg xylazine and 0.08 mg/kg atropine). Heart tissues were collected before ischemia (I-0H) and at various time points after reperfusion: 0H, 2H, 6H, 1 day, 3 days, and 7 days^48^ Cardiac apex tissue samples from mice were utilized for analysis. During the ischemic phase, long-chain acylcarnitine (L-Palmitoylcarnitine chloride, 100 mg/kg, intraperitoneal injection, single dose) and the CPT1A inhibitor etomoxir (ETO, 50 mg/kg, intraperitoneal injection, single dose) were administered via intraperitoneal injection to mice. These mice subsequently underwent reperfusion for either 6 h (R-6H) or 7 days (R-7D) to induce injury.^49^^,^^50^ All experimental procedures were approved by the Animal Care Committee of Capital Medical University and conducted in accordance with the Animal Management Rules of the Chinese Ministry of Health (Document No. 55, 2001) as well as the Directive 2010/63/EU of the European Parliament and the NIH Guide for the Care and Use of Laboratory Animals. The influence of sex on the study results was not specifically evaluated and thus represents a limitation of this work.

#### Cell culture and treatment

Human umbilical vein endothelial cells (HUVECs) were purchased from ScienCell Research Laboratories (ScienCell, 8000) and cultured in endothelial cell medium (ScienCell) containing 5% (vol/vol) fetal bovine serum (FBS) and 1% (vol/vol) endothelial cell growth supplement. The HUVECs were tested for mycoplasma contamination upon receipt and were confirmed to be negative. Mouse neonatal cardiomyocytes (NCMs) were isolated according to our previously published study and cultured in cardiomyocyte medium (ScienCell) containing 5% (vol/vol) FBS and 1% (vol/vol) cardiac myocyte growth supplement.^51^ Adult rat cardiomyocytes (ACMs) were isolated as previously described and cultured in Dulbecco’s Modified Eagle Medium (ScienCell) containing 5% (vol/vol) FBS.^52^

A parallel-plate flow system was used to impose shear stress on HUVECs or NCMs cultured in flow channels using established methods.^53^ High laminar fluid shear stress (FSS) of 25 dyn/cm^2^ for 4 h was applied to HUVECs or NCMs.^54^

HUVECs were treated with etomoxir (40uM, MCE, HY-50202) or CPT1A siRNA for 10 or 48 h before fluid shear stress testing.^55^ The siRNAs used to knock down CPT1A in HUVECs were synthesized by RiboBio (Guangzhou, China). The efficiency of siRNA knockdown was evaluated by quantitative PCR (qPCR) (Figure S4C). For qPCR primers see (Table S5).

ACMs and NCMs were serum-starved overnight and divided into four groups. They were then treated with palmitoyl-L-carnitine (LCAC C16) (P1645, Sigma-Aldrich), at concentrations of 0, 50, 100, and 150 μM, respectively, for 4 h^56^ NCMs were serum-starved overnight and incubated with Meldonium (MET-88) (40 μM, MCE, HY-15409) for 12 h before LCAC C16 treatment.^57^

To induce hypoxia/reoxygenation, HUVECs or NCMs were subjected to hypoxia for 6 h and then reoxygenated for 12, 24 or 36 h.^58^ Anoxia experiments were carried out by placing the HUVECs in an anoxic incubator for 4 h (95% N2 and 5% CO2).^59^ For hypoxia conditions were achieved using the AnaeroPack system (Mitsubishi Gas Chemical, Tokyo, Japan).^60^

For the co-culture experiment, the HUVEC culture medium was replaced with FluoroBrite Dulbecco’s Modified Eagle’s Medium (#119955, Gibco) overnight, and FSS was applied as described previously. After 4 h of FSS treatment, the conditioned medium was collected and used to culture ACMs or NCMs for another 24 h.

### Method details

#### Blood samples collection

Peripheral blood samples were collected from all patients on the day of admission prior to revascularization, with an additional 5 samples obtained from 5 patients at 24 h post-procedure (*n* = 421). All peripheral blood samples were collected from patients in a fasted state (typically overnight for 8–12 h).Whole blood was separated at 4°C by centrifugation at 1,600 × g for 10 min. Plasma samples were stored at −80°C.

#### Targeted metabolic profiling

Targeted metabolic profiling of plasma samples was performed using the MxP Quant 500 targeted metabolomics kit (Biocrates Life Sciences AG, Innsbruck, Austria) as described previously.^61^ Plasma samples were processed for analysis as recommended by the kit manufacturer. Briefly, after being allowed to thaw and equilibrate to room temperature, samples were homogenized. 10-μL plasma aliquots, calibrators and controls were pipetted into the respective slots of a 96-well deep well reaction plate. The plate was dried for 30 min under nitrogen 5.0. Derivatization was performed by adding 50 μL 5% PITC prepared in a mixture of ethanol, pyridine and water (1:1:1, v/v) to each slot, covering and incubating the plate for 60 min at ambient temperature and, after removing the plastic lid, by drying for 60 min under nitrogen. 300 μL 5 mmol/L ammonium acetate was subsequently added and the plate was shaken on an Allsheng MD-200 plate shaker at 450 rpm, ambient temperature, for 30 min. Elution of the analytes into a 96-well deep-well collection plate was performed by applying positive pressure on a Phenomenex Presston manifold (Gen-Lab Kft., Budapest, Hungary). For runs including chromatographic separation, 150 μL extract was pipetted to an LC collection plate and was diluted with 150 μL water. For flow injection analysis, 10 μL extract was transferred to a FIA collection plate and was diluted with 490 μL mobile phase employed for the FIA runs.

#### PGC-1α assay

PGC-1α levels in patient serum plasma and swine heart tissue were measured by ELISA using the PGC-1α assay kit (F11500-B, Fankew, Shanghai, China) according to the manufacturer’s protocol. Reagents and samples were mixed and immediately reacted at 37°C for 30 min in the dark, followed by transferring liquid to a 1 mL glass cuvette, and the absorbance value was read at 450 nm. The concentration of PGC-1α was calculated according to a standard curve obtained.

#### Cardiac troponin I assay

Porcine cardiac troponin I (cTnI) levels in decanted plasma were measured using a standard ELISA kit (Kamiya Biomedical, Seattle, WA) with internal standards. Plasma samples were diluted with three volumes of a specific diluent. Subsequently, calibrators and diluted samples were incubated in microtiter wells with horseradish peroxidase (HRP) conjugate for one hour. During this step, cTnI molecules are captured in a sandwich complex between immobilized antibodies and the detection antibodies. After incubation, the wells were washed to remove any unbound HRP conjugate. Tetramethylbenzidine (TMB) substrate was then added and incubated for 20 min, resulting in the development of a blue color in the presence of cTnI. The color reaction was stopped by adding an acid stop solution, which changed the color from blue to yellow. The absorbance of the resulting solution was measured at 450 nm. The concentration of cTnI, which is directly proportional to the absorbance, was calculated from a calibration curve derived from the standards.

#### MDA assay

Malondialdehyde (MDA) contents in the myocardium were assayed following the method of MDA content test kit (Solarbio, Beijing, China). Briefly, tissues were homogenized in cell lysis buffer at 4°C. After centrifuging, the supernatant was mixed with thiobarbituric acid (TBA) working solution, and then incubated at 100°C for 15 min. After cooling, sample absorbance was measured at 532 nm using the microplate reader.

#### ATP assay

The ATP content in both cell and heart tissue samples was measured with a commercial ATP assay kit (Beyotime Biotechnology, China) according to the manufacturer’s instructions. For cell samples, lysis was performed using the provided lysis buffer, followed by centrifugation at 10,000 × g for 5 min at 4°C to collect the supernatant. For heart tissue samples, lysis buffer was added in a ratio of approximately 100–200 μL per 20 mg of tissue and homogenized thoroughly using a glass homogenizer to ensure complete lysis. The homogenate was then centrifuged at 12,000 × g for 5 min at 4°C, and the resulting supernatant was collected.Based on an ATP concentration-versus-absorptance standard curve, the ATP concentration was calculated.

#### NAD+/NADH assay

NAD^+^ and NADH levels in cell and heart tissue samples were measured using a commercial NAD^+^/NADH assay kit (Beyotime Biotechnology, China). Briefly, samples were lysed with 400 μL of the provided lysis buffer and centrifuged at 12,000 × g for 10 min. The resulting supernatant was collected for the assay.The assay procedure was as follows: First, 90 μL of alcohol dehydrogenase was aliquoted into a 96-well plate. To measure total NAD (NAD^+^ + NADH), 20 μL of the supernatant or standard was added directly to the wells. To specifically measure NADH, a separate 20 μL aliquot of the supernatant or standard was first incubated at 60°C for 30 min to decompose NAD^+^, and was then added to the plate. Subsequently, 10 μL of chromogenic solution was added to all wells, followed by a 30-min incubation at 37°C. The absorbance was measured at 450 nm using a multimode microplate reader. A standard curve was generated from the standards, and the NAD^+^ concentration was calculated by subtracting the NADH value from the total NAD value. The final NAD^+^/NADH levels were normalized to the total protein concentration of each sample, which was determined using a BCA protein assay.

#### LCACs analyses

Analysis acyl-carnitines was carried out at LipidALL Technologies as previously described.^62^ Briefly, 300 μL of extraction buffer containing isopropanol, 50 mM KH2PO4, 50 mg/mL BSA (25:25:1 v/v/v) acidified with glacial acetic acid was added to samples. Next, d3-16:0-carnitine was added as internal standards and lipids were extracted by incubation at 4°C for 1 h at 1500 rpm. Following this, 300 μL of petroleum ether was added and the sample was centrifuged at 12000 rpm for 2 min at 4°C. The upper phase was removed. The samples were extracted two more times with petroleum ether as described above. To the lower phase finally remaining, 5 μL of saturated ammonium sulfate was added followed by 600 μL of chloroform:methanol (1:2 v/v). The sample was then incubated on a thermomixer at 450 rpm for 20 min at 25°C, followed by centrifugation at 12000 rpm for 5 min at 4°C. Clean supernatant containing long-chain acyl-carnitines was transferred to fresh tube and subsequently dried in the SpeedVac under OH mode (Genevac). The extract was resuspended in methanol:water (9:1 v/v) containing 0.05% acetic acid, and analyzed on a Shimadzu 30AD-UPLC coupled to Sciex Triple Quad 6500+.

#### Immunohistochemistry and immunofluorescence

Cardiac tissues were fixed in 4% paraformaldehyde in phosphate-buffered saline, embedded in paraffin, and sectioned at a thickness of 5 μm. The Fixation/Permeabilization Solution Kit (BD Biosciences) was used for cell fixation and permeabilization. ImageJ software was used to quantify positive staining. Cardiac apoptosis was determined using TUNEL staining, according to the manufacturer’s instructions (R&D Systems). Cardiac tissues and endothelial cells were immunostained with antibodies specific for CD31 (1:200, Abcam, Cambridge, UK), CPT1A (1:100, Abcam, Cambridge, UK) and CTNT (1:100, Proteintech, Wuhan, China). Nuclei were visualized with DAPI staining. Cell coverage area was determined in captured images with a Zeiss confocal microscope and analyzed using ImageJ software (National Institutes of Health, Bethesda, MD, USA).

#### Western blotting

Western blotting was performed as described previously. Briefly, equal amounts of lysate were loaded on 10% or 8% sodium dodecyl sulfate-polyacrylamide gel electrophoresis gels and transferred onto polyvinylidene fluoride membranes (Millipore, WI, US). After blocking with 5% bovine serum albumin or 5% skimmed milk, the membranes were incubated with the appropriate primary antibodies overnight at 4°C, followed by incubation with anti-rabbit immunoglobulin G or anti-mouse immunoglobulin G horseradish peroxidase antibody (1:2000, Cell Signaling Technology) as secondary antibodies. Then, immunoreactive proteins were visualized using an electrochemiluminescence system (GE Healthcare Biosciences, Pittsburgh, PA, US). GAPDH were used as loading controls. Quantification of Western blots was performed using ImageJ software (US National Institutes of Health). Primary antibodies against the following targets were used: CPT1A (1:1000, Abcam), OCTN2 (1:1000, Proteintech), GAPDH (1:1000, Abcam).

#### Cell viability and mitochondrial function assay

Cell viability and mitochondrial membrane potential were determined using a Mitochondrial Membrane Potential Detection Kit (C1071; Beyotime, Shanghai, China).^63^ The cells were harvested by centrifugation for 5 min at 1,000 *g* and resuspended in 50 mM phosphate-buffered saline (pH 7.0). A total of 50,000 cells collected by centrifugation were resuspended in 188 μL Annexin V-FITC. The mixture was prepared by adding 2 μL Mito-Tracker Red CMXRos and 5 μL Annexin V-FITC, before incubating for 30 min at 25°C and placing in an ice bath. Finally, sample smears were visualized under a Leica DMi8 fluorescence microscope (Leica Microsystems, Germany). The whole process was performed in the dark using aluminum foil.

Cell viability was evaluated using CCK8 (Beyotime Biotechnology, China). Cells (2 × 10^3^) were seeded in 96-well plates. CCK8 solution (20 μm L) was added to each well, and the plates were incubated at 37°C for 4 h. Absorbance was measured at 450 nm on a microplate reader.

The cell mitochondrial membrane potential was detected by the JC-1 mitochondrial membrane potential detection kit (Solarbio, Beijing, China). Cells were seeded in 6-well plates and stained for mitochondrial membrane potential assessment using the JC-1 fluorescent probe. Briefly, the culture medium was replaced with 500 μL of fresh medium containing 25 μM JC-1, followed by incubation at 37°C for 20 min. After staining, the cells were washed twice with warm phosphate-buffered saline and fixed with 2% paraformaldehyde. JC-1 fluorescence was quantified by flow cytometry (BD FACScalibur). Mitochondria with high membrane potential in healthy cells contain red fluorescent J-aggregates, which were detected in the FL-2 channel. In contrast, mitochondria with diminished membrane potential in apoptotic cells exhibit green fluorescent monomers, which were measured in the FL-1 channel.

#### Seahorse OCR analysis

The oxygen consumption rate (OCR) was measured using a Seahorse XFe96 analyser (Seahorse Bioscience). Isolated adult rat cardiomyocytes were differentiated in customized Seahorse 96-well plates and treated described above. After treatment, the medium was replaced with DEME Medium (Seahorse Bioscience), supplemented with 1 mM pyruvate, 2 mM glutamine, and 10 mM D-glucose. Measurements were taken as the cells were incubated sequentially under four conditions: (1) basal levels were measured with no additives; (2) oligomycin (1.5 μM) was added to reversibly inhibit ATP synthase and OXPHOS, showing glycolysis alone; (3) FCCP (1 μM), a mitochondrial uncoupler, was added to induce maximal respiration; and (4) Antimycin A (10 μM), a Complex I inhibitor and mitochondrial poison, was added to end the reaction. The Seahorse software was used to plot the results. OCR was normalized to cell numbers per well.^64^

#### Transmission electron microscopy

Freshly isolated hearts were fixed with 2.5% glutaraldehyde in PBS (pH 7.0) at 4°C for 8 h and then post-fixed for 1 h in 1% O_s_O_4_. After being dehydrated and embedded, tissues were cut into ultrathin sections (70–90nm) using an ultramicrotome and stained with uranyl and lead citrate. Mitochondrial observation and image acquisition were performed using a transmission electron microscope (model #HT-7800; Hitachi, Japan).

#### Echocardiography

After hair removal from the thoracic region, mice were anesthetized with 1–2% inhaled isoflurane and positioned in the supine position on a physiological monitoring platform. High-resolution imaging was performed using the Vevo imaging system. B-mode images were acquired along the left ventricular minor axis. From the mid-ventricular short-axis view, two-dimensional measurements were taken to determine the thickness and internal diameter of the anterior and posterior walls during both diastole and systole.

#### Morphological analysis

Morphological assessment was performed using hematoxylin and eosin (H&E), Masson’s trichrome, and wheat germ agglutinin (WGA) staining. H&E and Masson’s trichrome staining were used to evaluate overall tissue morphology and ventricular fibrosis, respectively. Cardiomyocyte cross-sectional area was assessed using WGA staining (Sigma-Aldrich).All images were acquired using an Olympus VS200 slide scanner and visualized with CaseViewer software.

#### Peptide identification and protein quantification

Peptide identification and protein quantification were performed in MaxQuant. Raw files were searched against the human National Center for Biotechnology Information (NCBI) Refseq protein database by Mascot 2.3 (Matrix Science Inc) implemented on Proteome Discoverer 1.4 (Thermo Scientific). The mass tolerances were 20 ppm for precursor and 50 mmu for product ions from Q Exactive Plus and Q-Exactive HF, and 20 ppm for precursor and 0.5 Da for productions for Fusion and Q-Exactive HF, respectively. Up to two missed cleavages wereallowed. The search engine set cysteine carbamidomethylation as a fixed modification and N-acetylation, oxidation of methionine as variable modifications. Precursor ion score charges were limited to +2, +3, and +4. The data were also searched against a decoy database so that protein identifications were accepted at afalse discovery rate of 1%. Label-free protein quantifications were calculated using a label-free, intensity-based absolute quantification (iBAQ) approach. FOTdefined as a protein’s iBAQ divided by the total iBAQ of all identified proteins within one sample, was used to represent the normalized abundance of a particular protein across samples.^65^

#### Untargeted metabolomics

Untargeted metabolomics analysis was conducted by Calibra Scientific, Inc., Key Laboratory of Digital Technology of Zhejiang Province (Calibra Scientific, China) on its CalOmics platform. Samples were extracted using methanol in a ratio of 1:4. The mixtures were shaken for 3 min and precipitated by centrifugation at 4000 × g, 10 min at 20°C. Four aliquots of 100 μL supernatant were transferred to sample plates and dried under blowing nitrogen, then re-dissolved in reconstitution solutions for sample injection into UPLC-MS/MS systems. The instruments for the four UPLC-MS/MS methods are ACQUITY 2D UPLC (Waters, Milford, MA, USA) plus Q Exactive (QE) hybrid Quadrupole-Orbitrap mass spectrometer (Thermo Fisher Scientific, San Jose, USA). QE mass spectrometer was operated at a mass resolution of 35000, the scan range was 70–1000 m/z. Raw data pre-processing, peak finding and peak annotation were processed using an in-house developed software. There were eight steps of data extraction and analysis which included extracted ion chromatograms (EIC) extraction, EIC smoothing, EIC noise estimation, peak selection, peak integration, peak screening, peak identification, and batch optimization. Peaks with the following situations were excluded: apex intensity less than 3000, less than 7 scan points, RT width less than 0.02min, and a signal to noise ratio <3. Software approved peaks and related spectra were subjected to further manual inspection. Metabolites were identified by searching an in-house reference standard library, which contains thousands of entries derived from purified metabolite standards analyzed on the same LC-MS platform. This approach ensured that metabolite identifications met the Level 1 standard of the Metabolomics Standards Initiative (MSI) as defined by its Chemical Analysis Working Group (CAWG). Metabolite identification was based on three criteria: a narrow retention time window (deviation <0.1 min within an analytical run), accurate mass (deviation <10 ppm), and MS/MS spectral match (forward and reverse matching scores >75% compared to reference library entries). The peak area for each metabolite was calculated based on the area under the curve.

#### RNA sequencing and data analysis

Bulk RNA sequencing was conducted by Novogene Co., Ltd. (Beijing, China) using the Illumina platform. Briefly, mRNA was purified with poly-T oligo-attached magnetic beads, randomly fragmented, and was subjected to cDNA synthesis and library generation using an NEBNext Ultra RNA Library Prep Kit. Library quality was assessed with q-PCR to quantify library effective concentration (>2 nM). The library was diluted to 1.5 ng/mL using Qubit2.0 (Invitrogen) results, and insert size was detected by an Agilent 2100 Bioanalyzer (Agilent). Sequencing was performed with an Illumina NovaSeq 6000 system (Illumina). Raw reads were filtered and mapped with STAR software.

Differentially expressed genes (DEGs) were identified using DESeq with a significance threshold of *p* < 0.05. Genes exhibiting a |log2(fold change)| > 1 and *p* < 0.05 were subsequently selected for Gene Ontology (GO) enrichment analysis, which was performed using the clusterProfiler package in R.For a comprehensive pathway-level interpretation, Gene Set Enrichment Analysis (GSEA) was conducted using software from the Broad Institute (http://software.broadinstitute.org/gsea). Additionally, Kyoto Encyclopedia of Genes and Genomes (KEGG) pathway enrichment analysis (https://www.kegg.jp/kegg/) was applied to the set of differentially expressed genes.

#### Interaction network analysis

Edge weights were calculated as pairwise Pearson’s correlation coefficients for every two nodes, and only edges with an absolute weight ≥0.7 are shown in the plots.^66^ Module analysis of the protein–protein interaction network was performed using Cytoscape software (version 3.9.1; Cytoscape Consortium), and plugin MCODE was used to identify the related nodes.

#### Molecular docking

The chemical structures of palmitoylcarnitine and acetyl-L-carnitine were downloaded from the PubChem database. The 3D structure of OCTN2 was retrieved from AlphaFold (https://alphafold.ebi.ac.uk/entry/O76082). Molecular docking was performed using AutoDock Vina.^67^ During molecular docking, water molecules were eliminated, and polar hydrogens were added to the protein structure to simulate the intermolecular interactions between the OCTN2 protein and the ligand at the active site.

### Quantification and statistical analysis

All statistical analyses were performed using R, GraphPad Prism, and IBM SPSS Statistics version 22.0 (IBM Corp., Armonk, NY, USA).

For clinical parameters, normally distributed continuous variables are presented as mean ± standard deviation (SD) and were compared using the unpaired Student’s *t* test. Non-normally distributed data are presented as median with interquartile range. Categorical variables, described as frequencies (percentages), were compared using Fisher’s exact test. Univariate and multivariate Cox proportional hazards models were used to identify risk factors affecting clinical outcomes. The Kaplan-Meier method was used to plot survival curves, and the log rank test was employed for comparison. The area under the receiver operating characteristic curve (AUC) was used to quantify predictive performance.

Both metabolomic and proteomic data were log2-transformed and median-normalized prior to analysis. Differential expression analyses for proteins and metabolites were performed using the limma R package.^68^ Correlations between LCAC C16:1 levels and clinical parameters were assessed using Spearman’s correlation coefficient. In network plots, edge weights were defined as pairwise Pearson correlation coefficients. Only edges with an absolute weight ≥0.7 are displayed, with their widths proportional to the corresponding weights. The graph layout was generated using multidimensional scaling, where the distance between two nodes is inversely proportional to their pairwise Pearson correlation coefficient.

Statistical details for each experiment, including the statistical tests used, the exact value of n, and a description of what n represents, are provided in the corresponding figure legends. A *p*-value <0.05 was considered statistically significant, and significance levels are denoted by asterisks: ∗*p* < 0.05, ∗∗*p* < 0.01, and ∗∗∗*p* < 0.001.

## Supplemental information

Document S1. Figures S1–S8 and Tables S1–S5

Document S2. Article plus supplemental information
