# Supplementary material for: Targeting long-chain acylcarnitine accumulation to protect cardiac mitochondrial homeostasis after complete revascularization
Source: Cell Rep Med. 2025 Dec 16;6(12):102507. doi: 10.1016/j.xcrm.2025.102507 (PMC12765950; doi:10.1016/j.xcrm.2025.102507)
Supplement: Document S1. Figures S1–S8 and Tables S1–S5 [file mmc1.pdf]

Cell Reports Medicine, Volume *6*

Supplemental information

Targeting long-chain acylcarnitine accumulation to protect cardiac mitochondrial homeostasis after complete revascularization

Rui Lin, Yuyu Li, Shiwei Yang, Hai Gao, Fengjuan Li, Xue Wang, Xin Tan, Zhengkai Wang, Weiyao Chen, Lu Ren, Xiujie Wang, Li Wang, Jun Qin, Wenjie Yin, Jie Du, and Yuan Wang

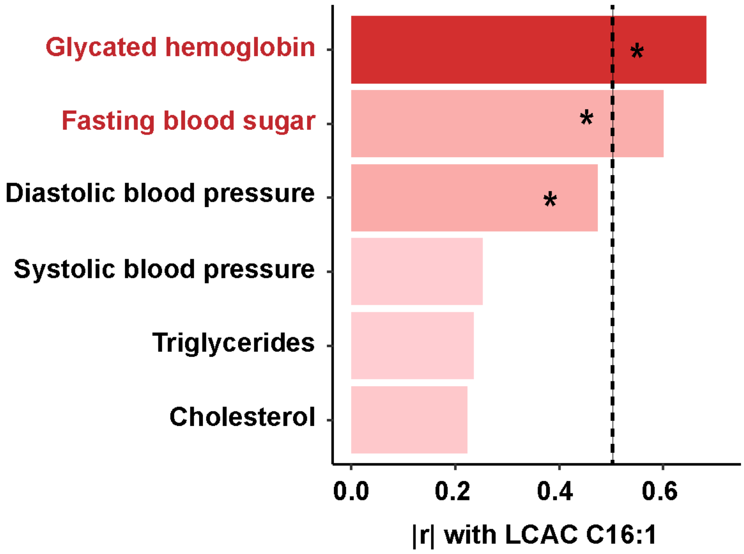

Figure S1. Correlation of the LCAC C16:1 with clinical parameters

Correlation of the LCAC C16:1 with clinical parameters (glycated hemoglobin, fasting blood sugar, diastolic blood pressure, systolic blood pressure, Triglyceride and Cholesterol). *,p<0.05, Spearman correlation analysis.

LCAC, long-chain acylcarnitine.

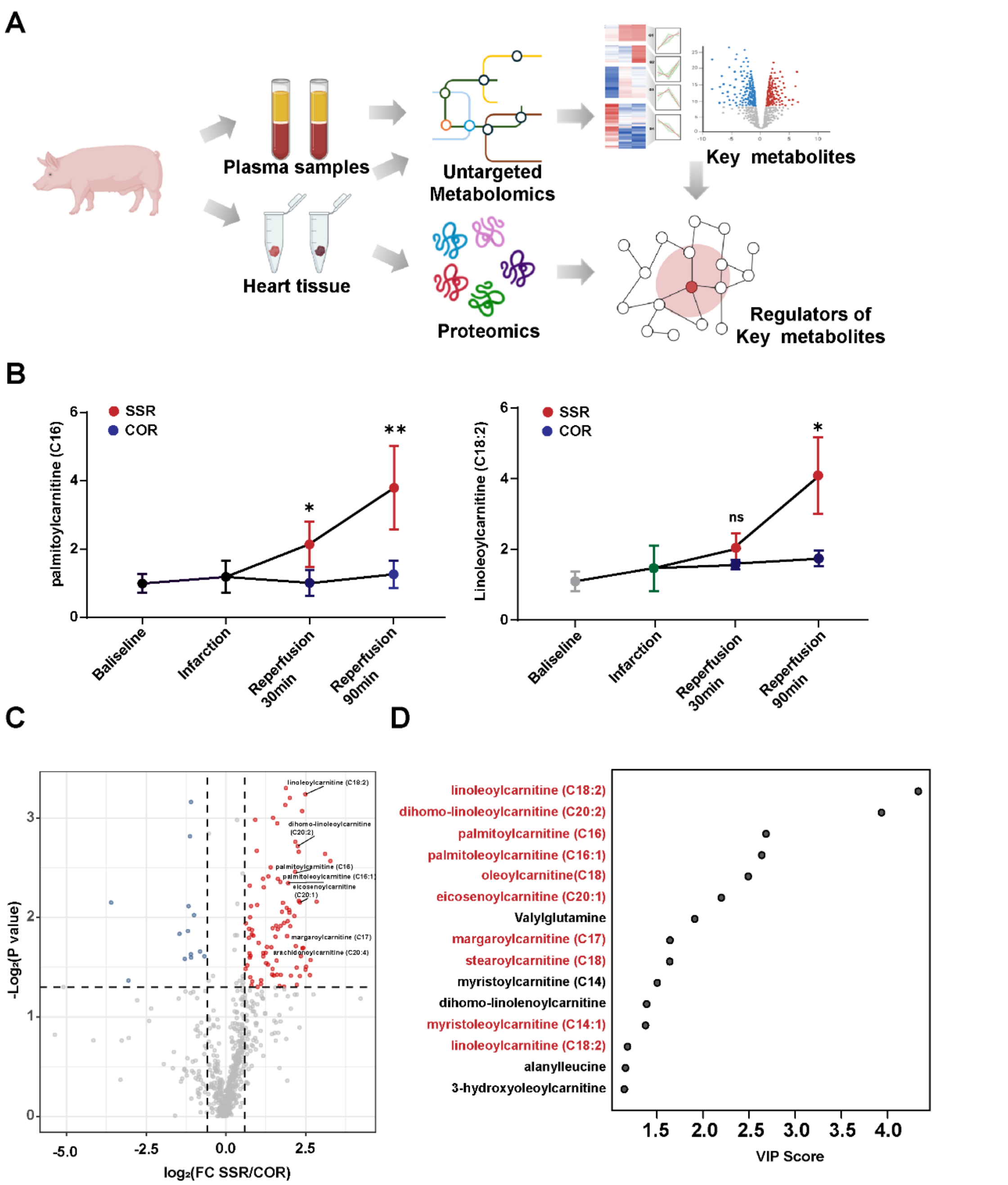

Figure S2. Multi-omics Experimental Design and Differential Metabolite Analysis in SSR and COR Groups

(A) Schematic showing the multi-omics based experimental design using cardiac vein plasma samples and heart tissue.

(B) Coronary vein levels of LCAC C16 and C18:2 in the baseline, infarction 30, after 30 and 90 min of reperfusion in SSR and COR (n=6/group). Data were presented as means ± standard deviations as indicated. **p* < 0.05 and ***p* < 0.01 in a Student's unpaired t test.

(C) Volcano plot of differential metabolites (SSR vs COR) in cardiac vein (n=6/group).

(D) Weight analysis of differential metabolites in the OPLS-DA model.

SSR, single-stage revascularization group; COR, culprit-only revascularization group; LCAC, long-chain acylcarnitine; FC, Fold Change; VIP, variable importance.

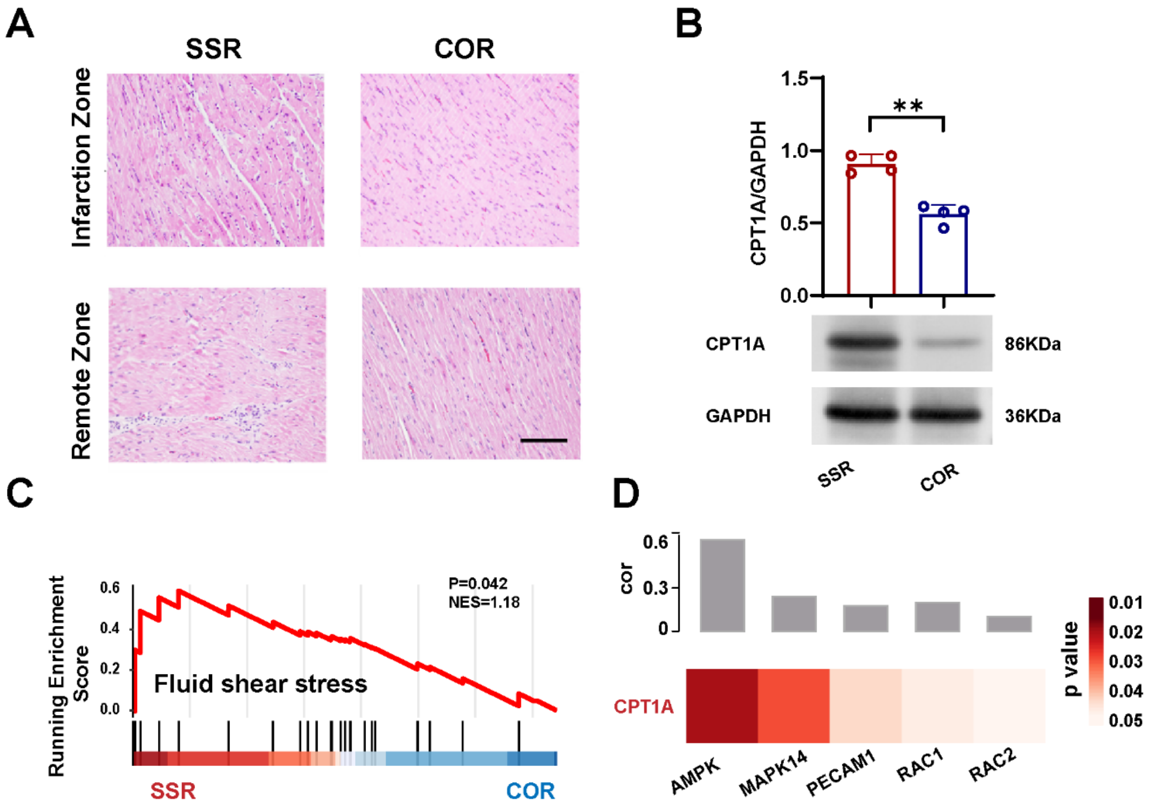

Figure S3. CPT1A Upregulation in SSR is Associated with the Fluid Shear Stress Signaling Pathway

(A) Representative photomicrographs of hematoxylin and eosin staining showing histological changes in cardiac tissues from swine. Scale bar: 200 µm.

(B) Protein CPT1A expression quantitation in the COR and SSR groups and respective quantification (n=4/group). Data were presented as means ± standard deviations as indicated. **$p < 0.01$ in a Student's unpaired t test.

(C) GSEA analysis of Fluid Shear Stress pathway.

(D) Correlation analysis of FSS-related proteins (PECAM1, AMPK, MAPK14, RAC1, and RAC2) and CPT1A by spearman correlation analysis.

SSR, single-stage revascularization group; COR, culprit-only revascularization group;

cor, correlation coefficient; GSEA, Gene Set Enrichment Analysis; NES, normalized enrichment score; CPT1A, carnitine palmitoyltransferase 1A; FSS, fluid shear stress.

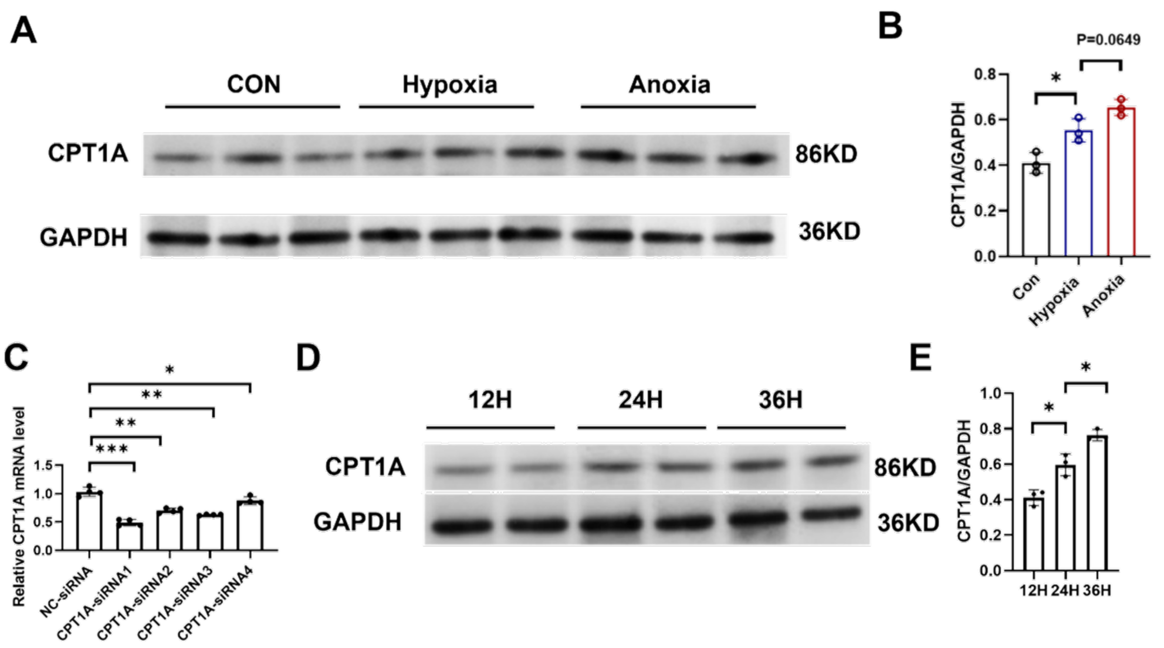

Figure S4. CPT1A Expression under Hypoxia and Reoxygenation and Validation of its siRNA Silencing

(A-B) Quantification of CPT1A protein expression in HUVECs treated under control, hypoxia, and anoxia conditions (n=3/group). Data were presented as means ± standard deviations as indicated. *$p < 0.05$ in a Student's unpaired t test.

(C) Relative CPT1A mRNA level in HUVECs treated NC-siRNA or CPT1A-siRNA (n=4/group).The non-targeted siRNA served as the negative control. *,p<0.05,**, $p < 0.01$,***, $p < 0.001$ in a Student's unpaired t test. CPT1A-siRNA 1 was detected with the highest silencing efficacy and used for subsequent experiments.

(D-E) Quantification of CPT1A protein expression in NCMs expression at 12, 24, and 36 hours after reoxygenation. (n=3/group). Data were presented as means ± standard deviations as indicated. *$p < 0.05$ in a Student's unpaired t test.

CPT1A, carnitine palmitoyltransferase 1A; NC, negative control; siRNA, silencing RNA; NCMs, mouse neonatal cardiomyocytes.

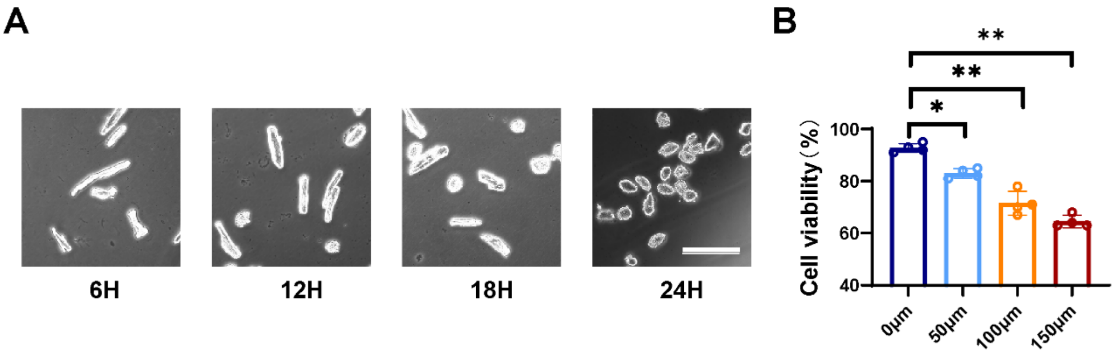

Figure S5. Cytotoxic Effects of LCAC on Adult Cardiomyocytes: Impaired Viability and Altered Morphology

(A)Morphological changes of ACMs co-cultured at different time points. Scale bar: 100 μm.

(B) CCK8 assay was used to determine the inhibition rate of ACMs treated various concentrations of LCAC (n=4/group). Data were presented as means ± standard deviations as indicated. *$p < 0.05$,**, $p < 0.01$ in a Student's unpaired t test.

ACMs, Adult rat cardiomyocytes

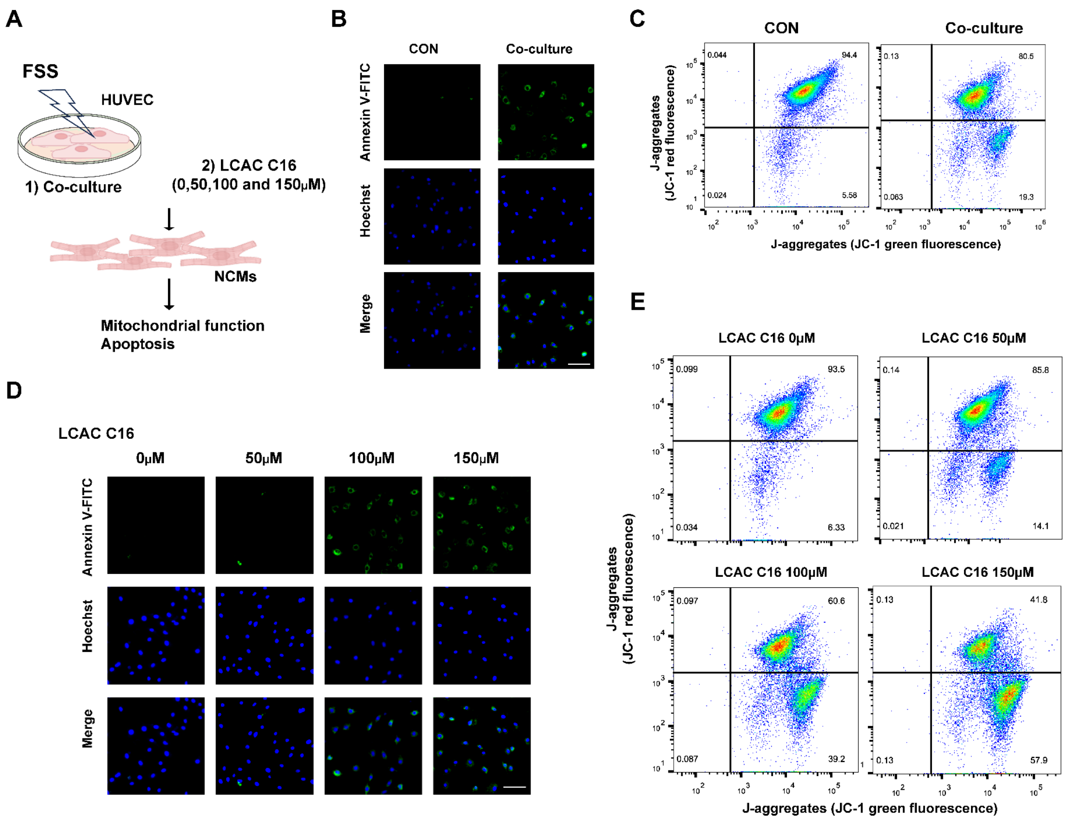

Figure S6. LCACs Trigger Cardiomyocyte Apoptosis and Disrupt Mitochondrial Function in NCMs

(A) Schematic of the in vitro experimental protocol, involving 1) co-culture of HUVECs subjected to FSS stimulation with NCMs and 2) exposure of neonatal mouse cardiomyocytes to various concentration gradients of LCACs for stimulation.

(B) Annexin V-FITC (green fluorescence) staining was used to detect apoptosis of NCMs from the co-culture and control groups. Scale bar: 100 μm.

(C) The mitochondrial membrane potential of NCMs from the co-culture and control groups was measured. Representative graphs of the flow cytometry analysis after incubation with JC-1.

(D) NCMs were treated with various concentration gradients of LCACs, and apoptosis was detected by annexin V-FITC (green fluorescence) staining. Scale bar: 100 μm.

(E) NCMs were treated with various concentration gradients of LCACs for stimulation, and the mitochondrial membrane potential was measured. Representative graphs of the flow cytometry analysis after incubation with JC-1.

CPT1A, carnitine palmitoyltransferase 1A; FSS, fluid shear stress; HUVECs, human umbilical vein endothelial cells; LCAC, long-chain acylcarnitine; NCMs, mouse neonatal cardiomyocytes.

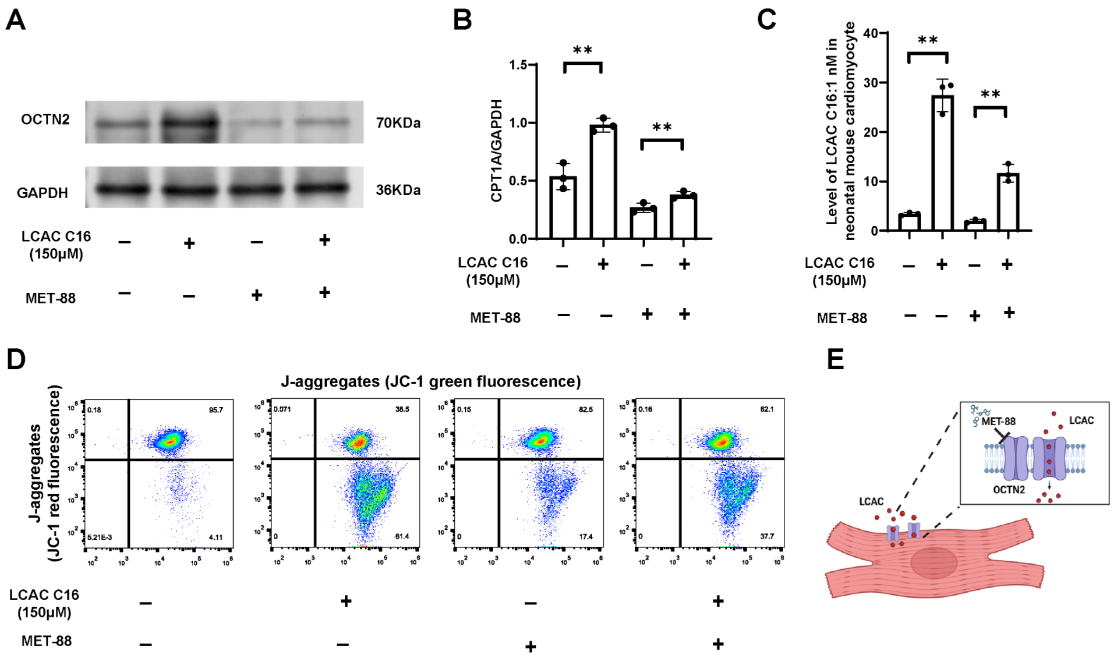

Figure S7. OCTN2 Mediates LCAC C16 Entry into Cardiomyocytes and Induces Mitochondrial Dysfunction

(A-B) OCTN2 protein expression in NCMs treated with LCAC C16 (150 μM) and MET-88(n=3/group). Data were presented as means ± standard deviations as indicated. **, $p < 0.01$ in a Student's unpaired t test.

(C) Level of LCAC C16:1 in NCMs treated with LCAC C16 (150 μM) and MET-88(n=3/group). Data were presented as means ± standard deviations as indicated. **, $p < 0.01$ in a Student's unpaired t test.

(D) The mitochondrial membrane potential in NCMs treated with LCAC C16 (150 μM) and MET-88. Representative graphs of the flow cytometry analysis after incubation with JC-1.

(E) Pattern diagram of LCACs entering myocardial cells through OCTN2.

LCAC, long-chain acylcarnitine; NCMs, mouse neonatal cardiomyocytes;OCTN2, type 2 organic cation transporter; MET, Meldonium.

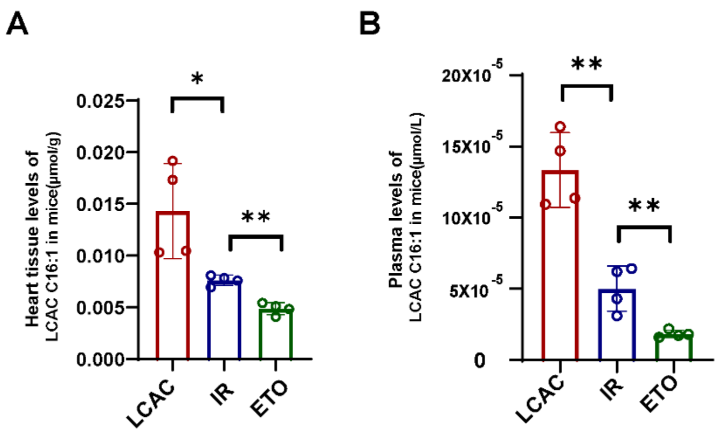

Figure S8. Comparison of LCAC C16:1 Levels in Heart and Plasma among Treatment Groups

(A-B)LCAC C16:1 of heart issue or plasma concentrations from the LCAC, IR, and ETO groups(n=5/group). Data were presented as means ± standard deviations as indicated. *,p<0.05,**, p < 0.01 in a Student's unpaired t test.

LCAC, long-chain acylcarnitine; ETO, etomoxir

Table S1: Baseline Characteristics of derivation cohort

	Non-MACEs (n=194)	MACEs (n=22)	*P*
Age, yr	59.86±9.16	60.47±10.60	0.902
Sex(male)—no. (%)	121(62.37)	13(59.09)	0.764
Diabetes—no. (%)	70(36.08)	13(59.09)	0.035
Hypertension — no. (%)	58(29.89)	11(50.00)	0.055
Hyperlipemia—no. (%)	57(29.38)	10(45.45)	0.122
GRACE score	116.24±14.24	129.40±12.15	0.059
SYNTAX score	16.57±6.81	16.01±6.33	0.226
SBP ,mmHg	132.56±13.14	141.51±11.20	0.051
DBP,mmHg	93.43±10.42	100.42±9.01	0.042
Glycated hemoglobin,%	16.21±2.30	19.13±9.12	0.034
Fasting blood sugar, mmol/L	6.42±1.92	9.43±2.13	0.063
Triglycerides, mmol/L	1.22±0.41	1.42±0.69	0.036
Cholesterol, mmol/L	4.52±9.01	6.19±10.42	0.041
Creatinine, umoI/L	83.00±4.02	91.21±5.12	0.047
hs-Tni */ after index PCI	1.27±0.27	1.66±0.26	0.332
BNP*/ after index PCI	2.12±0.38	2.31±0.32	0.305
LVEF/ after index PCI	53.32±4.02	48.29±5.25	0.047

Values are as means±standard deviation or n (%).

SBP, Systolic blood pressure; DBP, Diastolic blood pressure;BNP, brain natriuretic peptide;Tni, cardiac troponin I;LVEF, left ventricular ejection fraction.

*Data performed log transformation (base 10)

Table S2: Baseline Characteristics of validation cohort

	Non-MACEs (n=147)	MACEs (n=14)	*P*
Age, yr	57.86±9.16	59.47±10.60	0.865
Sex(male)—no. (%)	77(52.38)	7(50.00)	0.656
Diabetes—no. (%)	41(27.89)	8(57.14)	0.023
Hypertension — no. (%)	72(48.98)	7(50.00)	0.278
Hyperlipemia—no. (%)	29(19.72)	6(42.85)	0.045
GRACE score	119.24±14.24	128.40±12.15	0.042
SYNTAX score	16.57±6.81	17.01±5.33	0.036
SBP ,mmHg	131.53±13.12	135.52±8.29	0.051
DBP,mmHg	99.43±10.41	101.42±8.02	0.052
Glycated hemoglobin,%	16.25±2.31	19.16±9.17	0.034
Fasting blood sugar, mmol/L	6.43±1.91	7.43±2.26	0.163
Triglycerides, mmol/L	1.24±0.42	1.42±0.62	0.035
Cholesterol, mmol/L	4.55±9.01	6.18±10.41	0.043
Creatinine, umoI/L	84.21±4.09	91.23±5.12	0.058
hs-Tni */ after index PCI	1.24±0.17	1.64±0.21	0.331
BNP*/ after index PCI	2.12±0.35	2.31±0.31	0.302
LVEF/ after index PCI	53.43±3.03	48.43±5.23	0.042

Values are as means±standard deviation or n (%).

SBP, Systolic blood pressure; DBP, Diastolic blood pressure;BNP, brain natriuretic peptide;Tni, cardiac troponin I;LVEF, left ventricular ejection fraction.

*Data performed log transformation (base 10)

Table S3. Performance of SYNTAX score, GRACE score, or the combination of LCAC in predicting MACEs

	AUC	P value
SYNTAX score	0.724(0.675-0.773)	
SYNTAX score + LCAC C16:1	0.823(0.726-0.920)	<0.01
GRACE score	0.704(0.661-0.747)	
GRACE score +LCAC C16:1	0.795(0.719-0.871)	0.023

Table S4. Baseline characteristics of all patients

	Screening dataset (n=39)	Derivation cohort (n=216)	Validation cohort (n=161)
Age-yr	55.86±9.16	55.86±9.16	55.47±10.60
Sex(male)	27(69.23)	150(69.44)	105(65.22)
Diabetes	21(53.84)	114(52.78)	83(51.55)
Hypertension	23(58.97)	116(53.70)	108(67.01)
Hyperlipemia	17(43.59)	96(44.44)	71(44.10)
GRACE score	120.24±14.24	122.40±12.15	122.40±12.15
SYNTAX score	18.57±6.81	17.01±5.33	17.01±5.33
MACE	6(15.38)	36(16.67)	23(14.29)
Myocardial Injury	15(38.46)	92(42.59)	66(40.10)

Table S5. List of primers for qPCR used in this study

Gene (human)	Forward primer sequence	Reverse primer sequence
CPT1A	CAAACTGGACCGGGAGGAAA	TGTGCTGGATGGTGTCTGTC
GAPDH	GGTGGTCTCCTCTGACTTCAACA	GTTGCTGTAGCCAAATTCGTTGT
